# Single-cell characterization of monolayer cultured human dental pulp stem cells with enhanced differentiation capacity

**DOI:** 10.1038/s41368-021-00140-6

**Published:** 2021-12-15

**Authors:** Yu Cui, Wei Ji, Yongyan Gao, Yao Xiao, Huan Liu, Zhi Chen

**Affiliations:** 1 The State Key Laboratory Breeding Base of Basic Sciences of Stomatology, Key Laboratory of Oral Biomedicine, Ministry of Education (Hubei-MOST KLOS & KLOBM), School and Hospital of Stomatology, Wuhan University, Wuhan, China; 2 Department of Oral Implantology, School and Hospital of Stomatology, Wuhan University, Wuhan, China; 3 Department of Periodontology, School and Hospital of Stomatology, Wuhan University, Wuhan, China

**Keywords:** Stem-cell research, Stem-cell differentiation

## Abstract

Human dental pulp stem cells (hDPSCs) are easily obtained multipotent cells, however, their potential value in regenerative medicine is hindered by the phenotypic and functional changes after conventional monolayer expansion. Here, we employed single-cell RNA sequencing (scRNA-seq) to comprehensively study the transcriptional difference between the freshly isolated and monolayer cultured DPSCs. The cell cluster analysis based on our scRNA-seq data showed that monolayer culture resulted in a significant cellular composition switch compared to the freshly isolated DPSCs. However, one subpopulation, characterized as MCAM(+)JAG(+)PDGFRA(−), maintained the most transcriptional characteristics compared to their freshly isolated counterparts. Notably, immunofluorescent staining revealed that the MCAM(+)JAG(+)PDGFRA(−) hDPSCs uniquely located in the perivascular region of human dental pulp tissue. Flow-cytometry analysis confirmed that their proportion remained relatively stable (~2%) regardless of physiological senescence or dental caries. Consistent with the annotation of scRNA-seq data, MCAM(+)JAG(+)PDGFRA(−) hDPSCs showed higher proliferation capacity and enhanced in vitro multilineage differentiation potentials (osteogenic, chondrogenic and adipogenic) compared with their counterparts PDGFRA(+) subpopulation. Furthermore, the MCAM(+)JAG(+)PDGFRA(−) hDPSCs showed enhanced bone tissue formation and adipose tissue formation after 4-week subcutaneous implantation in nude mice. Taken together, our study for the first time revealed the cellular composition switch of monolayer cultured hDPSCs compared to the freshly isolated hDPSCs. After in vitro expansion, the MCAM(+)JAG(+)PDGFRA(−) subpopulation resembled the most transcriptional characteristics of fresh hDPSCs which may be beneficial for further tissue regeneration applications.

## Introduction

Human mesenchymal stem cells (MSCs) are multipotent cells, which exhibit a specific cell surface marker spectrum, and have multilineage differentiation potentials.^1^ MSC-like populations have been obtained and harvested from a variety of organs and tissues.^2^ Amongst them, dental pulp-derived MSCs (DPSCs), have been considered as one of the most promising cell source for maxillofacial tissue repair and regeneration owing to its abundance and ease of cell acquisition.^3–8^ Similar with other tissue-specific MSCs, the dental pulp-derived MSCs contain several subpopulations with different biological functions, which leads to a great heterogeneity. For instance, the heterogenous DPSC subpopulations have demonstrated differences in proliferation and differentiation potentials and proximally only two thirds DPSC subpopulations were able to form ectopic dentin in vivo, indicating their different regenerative capabilities.^3,9^

In order to identify distinct subsets of DPSC populations, researchers have optimized DPSC isolation based on a series of cell surface markers. DPSC is known to express MSC surface markers, such as CD29, CD44, CD59, CD73, CD90, and CD146, but no hematopoietic stem cell markers (e.g., CD14, CD34, CD45, and CD11b).^10^ Previous studies showed that DPSC subpopulations with different combinations of surface markers exhibited different tendency towards odontogenic, adipogenic and neurogenic commitment.^11,12^ In addition, DPSCs reside in the perivascular niche and the perineural niche in the postnatal dental pulp tissues.^13,14^ The intercellular communications between endothelial and stem cells within the perivascular niche influenced the self-renewing capacity of DPSCs,^15,16^ Furthermore, the superior neural and vascular properties of DPSCs opens their promising potentials for cell-based therapy of neurodegenerative and oral diseases.

Conventionally, DPSCs were isolated from the extracted tooth and expanded in vitro in monolayer prior to further cell-based applications.^3^ However, more and more evidences suggest that such monolayer expansion leads to an alteration of cell phenotype, evidenced^17^ by the CD44 expression and differentially expressed of CD73, CD106, CD146, and CD271. Furthermore, a recent study revealed that the DPSCs after monolayer expansion tend to lose multipotential properties,^17,18^ and continue with an unpredictable dynamic changes and functional variations of subpopulations.^19,20^ Several articles have reported the comprehensive view of the heterogeneity within the human dental pulp environment using single-cell RNA sequencing (scRNA-seq).^21–23^ However, up to date, the atlas of the monolayer expanded human dental pulp stem cells (hDPSCs) still remains unclear.

In view of this, the current study was aimed to comprehensively investigate the atlas of hDPSCs after 10-day monolayer expansion. We performed single-cell RNA sequencing of both freshly isolated and monolayer expanded DPSCs. Our data confirmed that the monolayer expansion induced an significant cellular composition shift, while only one subpopulation characterized as MCAM(+)JAG(+)PDGFRA(−) most resembles their fresh isolated state, resulting in enhanced differentiation potentials and elevated ectopic bone forming capacity. Combined, we believe these findings can improve reliability, reproducibility, and robustness of the hDPSCs for future applications in cellular therapy and tissue engineering.

## Results

### Cellular composition in human dental pulps

**Fig. 1:**
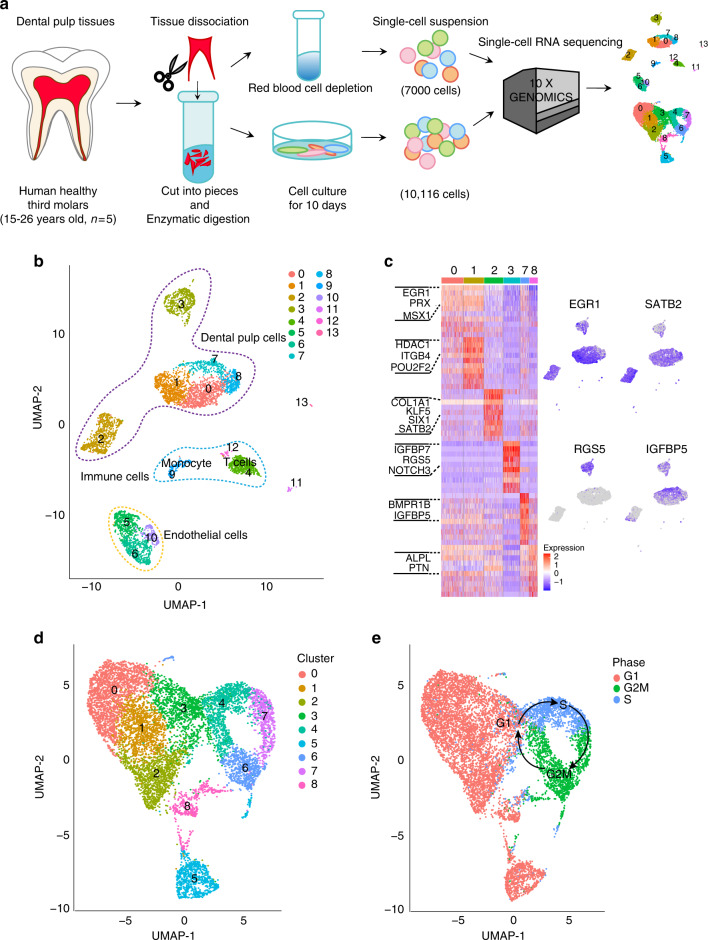
Single-cell characterization of freshly obtained and 10-day in vitro cultured human dental pulp stem cells (hDPSCs) by single-cell RNA sequencing (scRNA-seq). **a** The human dental pulp tissues were harvested from healthy third molars (age 15–25, *n* = 5), and cut into pieces followed by enzymatic digestion. After red blood cell depletion, the obtained freshly isolated cell suspension and the 10-day in vitro cultured cells were subject to single-cell RNA sequencing (scRNA-seq) using 10X Genomics, respectively. **b** Visualization of 14 color-coded clustering of the freshly isolated DPSCs (*n* = 6958 cells) using the Uniform manifold approximation and projection (UMAP) with SingleR annotations at the default resolution 0.5. Dots with the same color represents the cells assigned to the same cluster. The purple, blue and yellow dash lines indicated the populations of DPSCs (purple), immune cells (blue), and endothelia cells (yellow), respectively. **c** Heatmaps of the enriched genes identified from the freshly isolated human dental pulp cells (cluster 0, 1, 2, 7, 8) with representative marker genes expressions. **d** Visualization of eight color-coded clustering of the 10-day in vitro cultured DPSCs (*n* = 7500 cells) using the UMAP at the default resolution 0.5. **e** Cell-cycle phases of cultured hDPSCs. The red, blue and green dots represent the cell-cycle stage of G1 (red), S (blue), and G2/M (green). The arrows indicate cell-cycle directions: from G1 to S and then G2

To explore the cellular composition of human dental pulp tissues, we first used scRNA-seq to profile 7000 isolated cells from the fresh erythrocyte-depleted dental pulp tissues (Fig. 1a). The major cell populations were defined using the SingleR computational method^24^ to correlate single-cell gene expression with reference cell marker datasets of pure cell types recorded in the human cell atlas.^25^ As shown in Fig. 1b, the unbiased clustering showed 14 clusters, in which the dental pulp mesenchymal lineage (cluster 0, 1, 2, 3, 7, and 8) were clearly separated from the immune cells (cluster 4, 9, and 12) and endothelial cells (cluster 5, 6, and 10). Gene ontology analysis revealed that cluster 0 highly expressed genes associated with cell differentiation and skeletal system development, while cluster 1 expressed genes associated with cell–cell signaling and extracellular organization. In contrast, the genes enriched in cluster 7 and 8 were strongly related to neuron projection morphogenesis and ossification (Supplementary Fig. 1a).

We further assessed the uniquely-enriched marker genes expressed in the dental pulp mesenchymal lineage (Fig. 1c). Specifically, *VCAN, STAB2, SOX5,* and *GLI3* were highly expressed in cluster 2, which was strong related to blood vessel development and odontogenesis shown by gene ontology analysis. In addition, *RGS5, NOTCH3,* and *PDGFRB*, the perivascular cell markers, were exclusively expressed in cluster 3, while *POSTN* and *IGFBP5*, markers of the peri-odontoblastic layers,^21^ were highly expressed in cluster 7 (Fig. 1c and Supplementary Fig. 1b).

### Cellular composition in monolayer cultured hDPSCs

We also performed scRNA-seq to assess 10,116 monolayer cultured hDPSCs to identify their cellular composition. As shown in Fig. 1d, the monolayer cultured hDPSCs showed nine typical clusters (Fig. 1d), which were positive for classic MSC surface markers, including *ENG (CD105), NT5E (CD73), THY1 (CD90),* and *CD44* (Supplementary Fig. 2a), and negative for haemopoietic markers such as *PTPRC (CD45), CD34, CD14,* and *CD19* (Supplementary Fig. 2b).

**Fig. 2:**
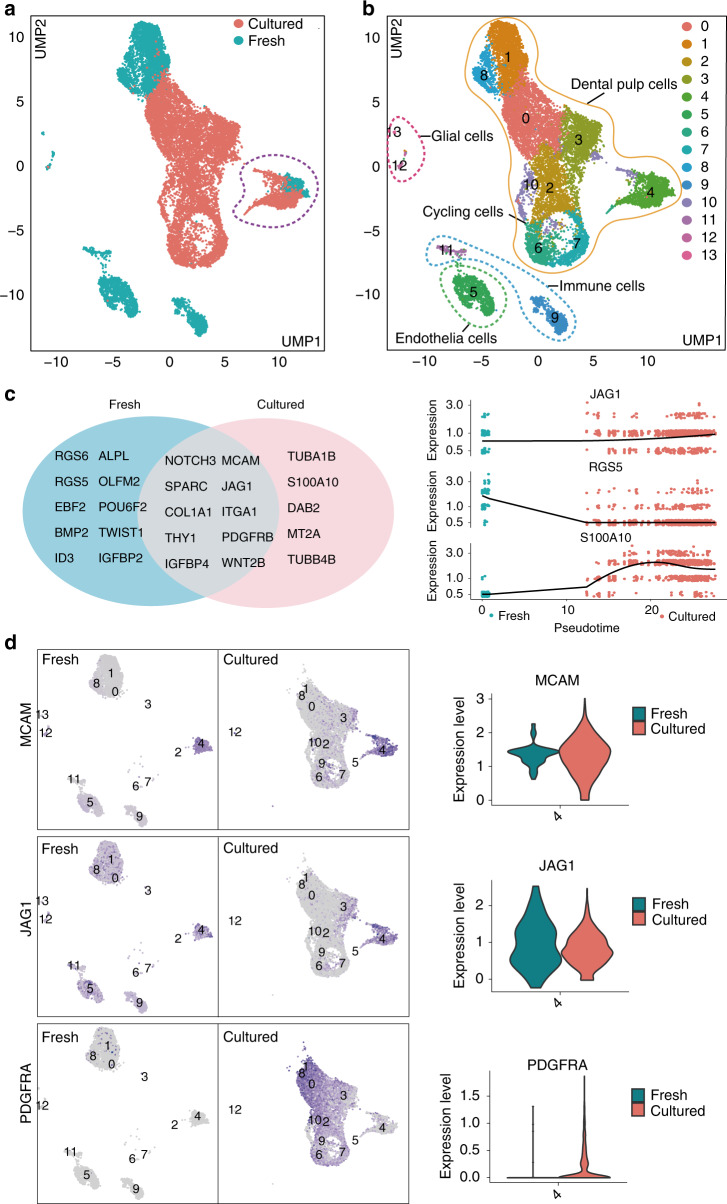
Cellular composition switch of freshly isolated human dental pulp stem cells (hDPSCs) upon in vitro culture. **a** UMAP visualization of monolayer cultured cells (red dots) compared to the freshly isolated cells (blue dots). The purple dash circles highlighted the most overlapped zone in both conditions. **b** UMAP visualization of 14 color-coded clusters of integrated freshly isolated and 10-day in vitro cultured hDPSCs. Four major cell types are identified, including dental pulp cells, endothelia cells, immune cells, and glial cells. Cluster 4 cells present in both datasets. **c** Differentially expressed genes in cluster 4 from fresh and cultured datasets were listed in the blue and pink circles, respectively. The overlapped gray region highlights the genes with similar expression levels in cluster 4 from both datasets. Expression dynamics along the pseudotime of selected genes from left panel was plotted by Monocle3. **d** The distribution (left) and quantification (right) of selected stem cell surface makers genes (MCAM, JAG1, and PDGFRA) expression in both cultured and fresh hDPSCs. Cells with low and high expression were marked with gray and purple color, respectively

Interestingly, we observed that cells in cluster 4, 6, and 7 formed a successive cell-cycle loop based on RNA velocity analysis (Supplementary Fig. 3b). Since the cell-cycle might influence gene expression, we further assigned each cell a cell-cycle phase according to a set of cell-cycle genes. Our data showed that Cluster 3, 4, 6, and 7 consisted of dividing cells assigned to the G2/M and S cell-cycle phases (Fig. 1e). For instance, Cluster 4 was enriched in S phases marker genes *CDCA7* and *MCM3* (Supplementary Fig. 4a); while Cluster 6 exhibited characteristic genes in the G2/M phase, such as *BIRC5* and *CCNB2* (Supplementary Fig. 4b).

GO enrichment assay further revealed that cells in different clusters showed different biological properties. Consistent with previously mentioned RNA velocity results, GO enrichment assay confirmed that cells in clusters 4, 6, and 7 highly expressed genes related to DNA replication, chromosome segregation, and cell-cycle process (Supplementary Fig. 4c). In comparison, cells in clusters 0, 1, and 2 highly expressed genes related to ossification, evidenced by high expressions of angiogenesis related genes such as C*XCL12*, *ANGPT1*, *IGFBP6*, and *FGF7* in cluster 0 and 1 cells and mineralized matrix organization related genes such as *COL1A2, COL5A3, ITGA11,* and *SPARC* in cluster 1 and 2 (Supplementary Fig. 5). Notably, cells in cluster 5 highly expressed perivascular cells or pericyte markers,^26^ such as *NG2 (CSPG4), PDGFRB, NOTCH3*, and *ACTA2* (Supplementary Fig. 6a). The enriched GO terms were associated with blood vessel development and cell differentiation, suggesting that cluster 5 might be perivascular niche cells with multilineage potentials in dental pulps (Supplementary Fig. 6b).

### Single-cell RNA sequencing reveals a cellular composition switch upon monolayer expansion

Next, we integrated the scRNA-seq profiles from fresh dental pulp and monolayer cultured hDPSCs and our data confirmed that the monolayer culture leads to a significant cellular composition alteration compared to the freshly isolated DPSCs (Fig. 2a). Cell composition across both datasets was visualized and 13 clusters was yielded, in which the cluster 4 was the most closely overlapping zone, suggesting that cluster 4 cells after monolayer most resembled their freshly isolated counterparts (Fig. 2b and Supplementary Fig. 7a).

We further assessed the differentially expressed genes enriched specifically in the cluster 4 cells from both the freshly isolated and cultured DPSCs. Our data showed that cluster 4 cells from both conditions expressed like *NOTCH3, THY1, MCAM, JAG1*, and *ITGA1*, the classic stem cell marker genes. However, *TUBA1B, S100A10* and *DAB2* were highly expressed in cultured samples whereas *RGS6, RGS5, EBF2*, and *POUF2* were highly expressed in fresh samples (Fig. 2c, Supplementary Fig. 7b, c). Furthermore, gene expression dynamics along the pseudotime revealed that after in vitro culture, *JAG1* was stably expressed and barely changed, while gene like *RGS5* and *S100A10* was down and upregulated, respectively, indicating the transcriptional changes still occurred during in vitro culture (Fig. 2c).

In addition, GO analysis on genes highly expressed in cluster 4 cells showed that biological process like cell morphogenesis involved in cell differentiation was enriched (Supplementary Fig. 8a). In consistence with the GO analysis result, we also found that the cluster 4 cells express classic MSC markers, like *ENG, NT5E, THY1, LEPR*, and *CXCL12*, but no *GLI1*. Furthermore, the expression of LEPR significantly declined during in vitro culture, while the expression of *ENG, NT5E*, and *CXCL12* showed significantly upregulated after monolayer expansion (Supplementary Fig. 10).

Interestingly, after monolayer expansion, the stem cell surface maker genes, *MCAM* and *JAG1*, were still highly expressed in cluster 4, whereas the *PDGFRA* was absent. Therefore, the cluster 4 cells after monolayer culture could be precisely selected as MCAM(+)JAG1(+)PDGFRA(−) subpopulation (Fig. 2d).

### The location of MCAM(+)JAG1(+)PDGFRA(−) cells in human dental pulp tissue and their resistance to aging and inflammation conditions

**Fig. 3:**
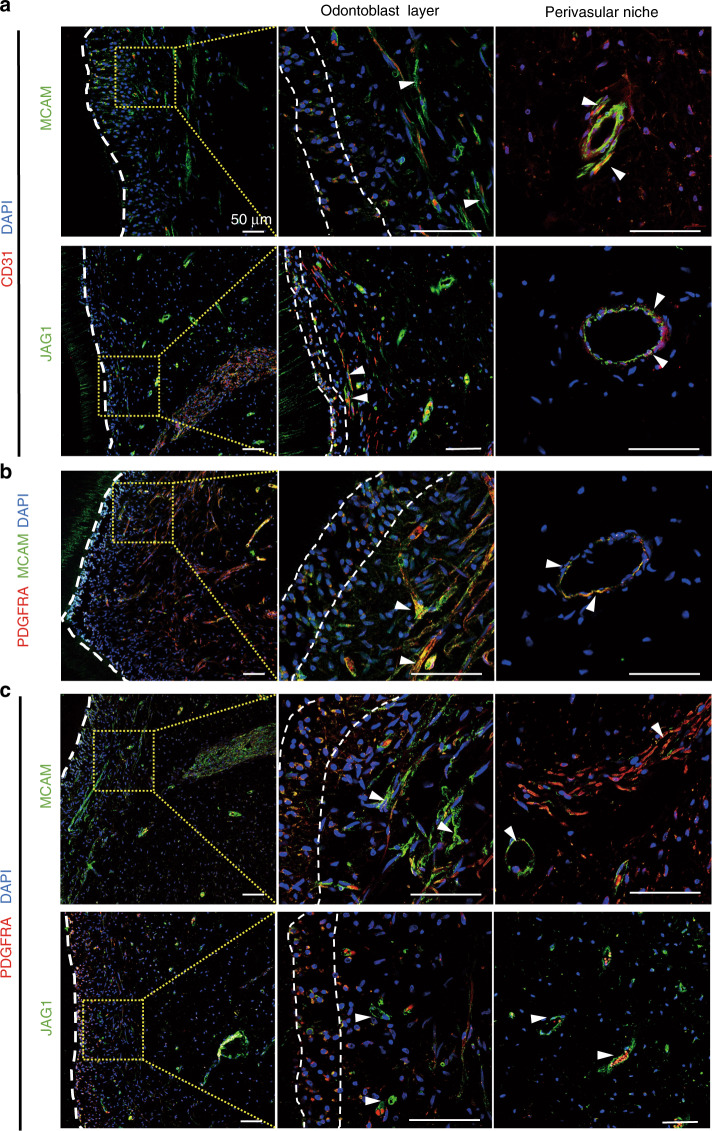
Distribution of MCAM(+)JAG1(+)PDGFRA(−) cells in the extracted human third molar. **a** Immunofluorescence double staining of endothelial cells markers CD31 (red) with MCAM (green, top) and JAG1 (green, bottom), respectively. **b** Immunofluorescence double staining of MCAM (red) and JAG1 (green) **c** Immunofluorescence double staining of PDGFRA (red) with MCAM (green) and JAG1 (green), respectively. The odontoblast layer was indicated by the dash lines, and the magnified area (dotted boxes) was shown in yellow dotted boxes (the middle panels). Nucleus were labeled with DAPI (blue). White arrowheads indicated characteristic blood vessel structures. Scale bars = 50 μm

Our next question was to identify the location of the MCAM(+)JAG1(+)PDGFRA(−) in the native healthy human dental pulp tissue. The immunofluorescent staining showed that cells positive in MCAM or JAG1 mainly located near blood vessels, as confirmed by co-staining with the vascular endothelial cell marker CD31 (Fig. 3a), and the MCAM and JAG1 simultaneously double-stained cells were mainly observed in the perivascular stem cell niches (Fig. 3b). On the other hand, PDGFRA was predominant within the odontoblastic layer and stroma, whereas the MCAM(+)JAG1(+)PDGFRA(−) cells were uniquely located in the perivascular region of human dental pulp tissue (Fig. 3c).

**Fig. 4:**
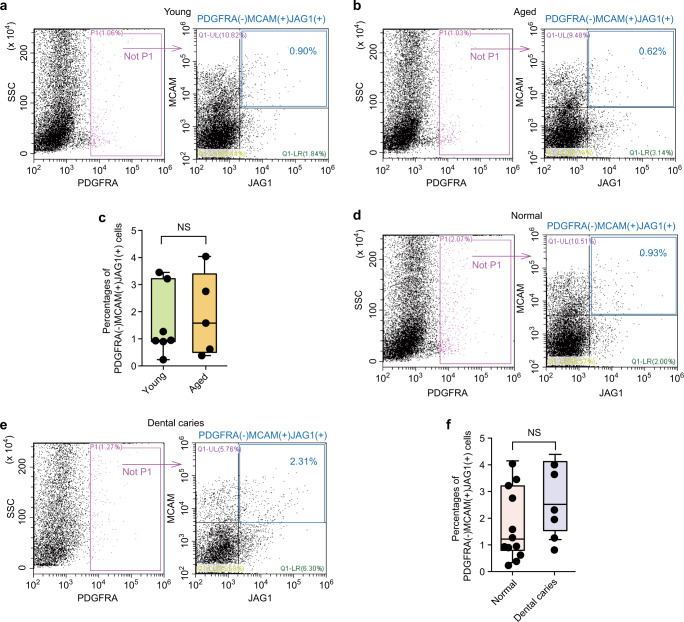
Flow-cytometry analysis of MCAM( + )JAG1( + ) PDGFRA(−) cells in human dental pulps from young, aged, healthy, and dental caries samples. **a** Representative graphs of dental pulp cell suspensions gained from young, following exclusion of PDGFRA(+) cells (purple boxes) and selection of double positive of JAG1 and MCAM cells (blue boxes). **b** Representative graphs of dental pulp cell suspensions gained from aged samples. **c** The percentage of MCAM(+)JAG1(+) PDGFRA(−) cells (blue numbers) in young/aged groups showed no significant differences by two-tailed *t* test. **d** Representative graphs of dental pulp cell suspensions gained from healthy samples. **e** Representative graphs of dental pulp cell suspensions gained from dental caries samples. **f** The percentage of MCAM(+)JAG1(+)PDGFRA(−) cells (blue numbers) in healthy and dental caries groups shows no significant differences by two-tailed *t* test

Interestingly, we also discovered that the expression of MCAM(+)JAG1(+)PDGFRA(−) hDPSCs in human dental pulps remained relatively stable regardless of physiological senescence or inflammation. FACS analysis revealed that the average percentage of MCAM(+)JAG1(+)PDGFRA(−) hDPSCs in young (age 15–24 years old) and aged (44–70 years old) dental pulps was 1.56% (range 0.23% to 3.45%) and 1.87% (range 0.38% to 4.04%) respectively, without statistical differences (Fig. 4a–c). Furthermore, the dental pulp derived from the decayed tooth showed 2.33% (range 0.81% to 3.64%) of MCAM(+)JAG1(+)PDGFRA(−) hDPSCs, which was comparable with that of 1.69% (range 0.23% to 4.04%) detected from the healthy dental pulp tissues (Fig. 4d–f). However, the proportion of MCAM(+)JAG1(+)PDGFRA(−) hDPSCs gradually reduced following in vitro culture for 1–4 passages (Supplementary Fig. 12).

### Biological performance of MCAM(+)JAG1(+)PDGFRA(−) subpopulation

**Fig. 5:**
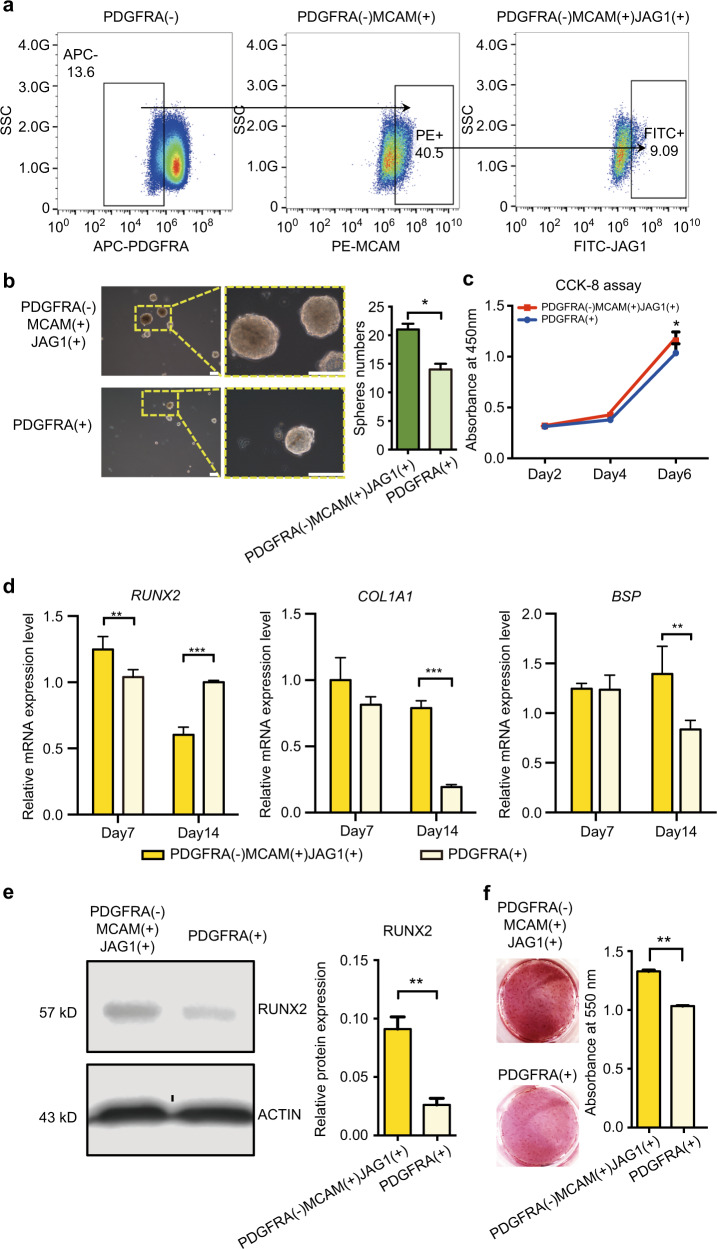
In vitro cellular performance of MCAM( + )JAG1( + ) PDGFRA(−) hDPSCs. **a** MCAM(+)JAG1(+) PDGFRA(−) and PDGFRA(+) hDPSCs were sorted by FACS. **b** Contrast microscopic images of cellular spheres (diameter exceeds 25 μm) formed by MCAM(+)JAG1(+) PDGFRA(−) and PDGFRA(+) hDPSCs, Scale bar = 100 μm; and the quantification of the spheres from each group formed after 7 days. *P < 0.05. **c** In vitro proliferation of MCAM(+)JAG1(+)PDGFRA(−) and PDGFRA(+) hDPSCs during 6-day culture. *P < 0.05. **d** Osteogenic marker genes expressions of *RUNX2, COL1A1*, and *BSP* of MCAM(+)JAG1(+)PDGFRA(−) and PDGFRA(+) hDPSCs after 14-day culture. **e** Western blot and quantitative analysis of RUNX2 expression in protein level after 7 days induction. **f** Alizarin Red S staining and quantification of the mineralized matrix obtained from MCAM(+)JAG1(+)PDGFRA(−) and PDGFRA(+) hDPSCs after 21 day culture in osteogenic medium

Next, we sorted MCAM(+)JAG1(+)PDGFRA(−) hDPSCs using flow cytometry to comprehensive investigate their biological performance (Fig. 5a). Compared with their counterparts PDGFRA(+), the MCAM(+)JAG1(+)PDGFRA(−) cells showed higher proliferation capacity, evidenced by significant larger and more sphere formation, and continue increase in cell number during 6-day in vitro culture (Fig. 5b, c).

In addition, the MCAM(+)JAG1(+)PDGFRA(−) cells showed an enhanced osteogenic differentiation potentials, evidenced by the significant upregulation of osteogenic lineage marker RUNX2 in both mRNA and protein level after 7-day osteogenic induction (Fig. 5d, e). After 14 days of induction, MCAM(+)JAG1(+)PDGFRA(−) cells showed a significant higher expression level of mineralized matrix-related genes *COL1A1* and *BSP* (Fig. 5d), consistent with the enhanced Alizarin red staining (Fig. 5f).

**Fig. 6:**
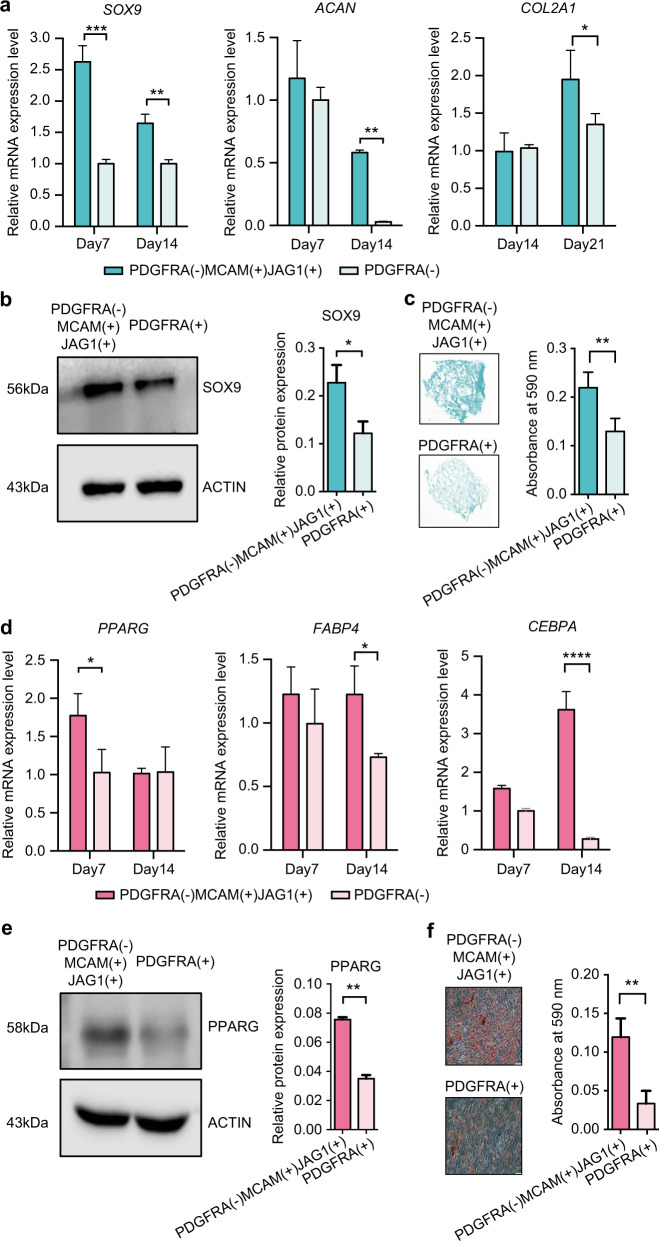
In vitro cellular performance of MCAM(+)JAG1(+) PDGFRA(−) hDPSCs. **a** Chondrogenic marker gene expression of *SOX9, ACAN,* and *COL2A1* of MCAM(+)JAG1(+)PDGFRA(−) and PDGFRA(+) hDPSCs after 14-day culture. **b** Western blot and quantitative analysis of SOX9 expression in protein level after 7 days induction. **c** Alcian blue staining and quantification of the cultured pellets obtained from MCAM(+)JAG1(+)PDGFRA(−) and PDGFRA(+) hDPSCs after 28 days induction. **d** Adipogenic marker gene expression of *PPARG, FABP4*, and *CEBPA* of MCAM(+)JAG1(+)PDGFRA(−) and PDGFRA(+) hDPSCs after 14-day culture. **e** Western blot and quantitative analysis of PPARG expression in protein level after 14 days induction. **f** Oil red O staining and quantification of the adipocytes obtained from MCAM(+)JAG1(+)PDGFRA(−) and PDGFRA(+) hDPSCs after 28 days induction. *GAPDH* was used as the normalization control in qPCR. The levels of ACTIN are used as loading control in western blot. Bars represent mean ± SD values. *P < 0.05; **P < 0.01; ***P < 0.001

We also found that MCAM(+)JAG1(+)PDGFRA(−) DPSCs showed an enhanced chondrogenic differentiation potentials than PDGFRA(+) cells, based on significant higher expression of. chondrocyte marker SOX9 in both mRNA and protein level at day 7 (Fig. 6a, b). The cartilaginous matrix-related gene *ACAN* and *COL2A1* were also upregulated in MCAM(+)JAG1(+)PDGFRA(−) cells after 14-day induction, which was consistent with higher glycosaminoglycan detected by Alcian blue staining (Fig. 6c). Similarly, MCAM(+)JAG1(+)PDGFRA(−) cells also showed higher adipogenic differentiation potentials, evidenced by higher expression of adipogenic markers such as *PPARG, FABP4*, and *CEBPA*, as well as more deposition of lipid droplets (Fig. 6d–f).

**Fig. 7:**
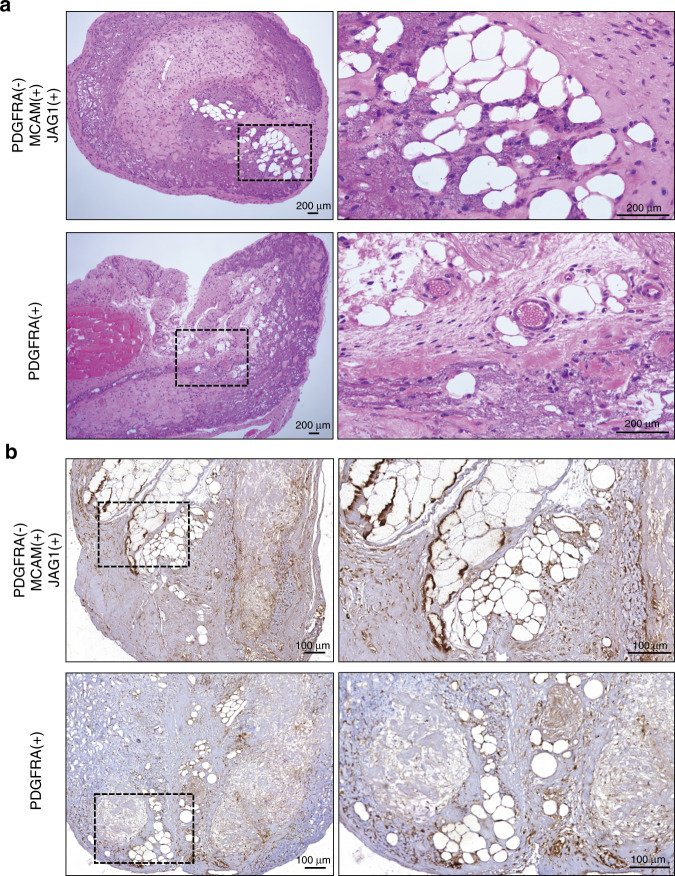
Adipogenic differentiation of MCAM( + )JAG1( + )PDGFRA(−) and PDGFRA( + ) hDPSCs in vivo. **a** Representative images of HE staining from cell-laden constructs when transplanted into immunocompromised mice for 4 weeks. A higher magnification emphasizing the yellow dotted portions. Scale bars = 200 μm. **b** Representative images of immunohistochemical staining of human specific mitochondria. Scale bars = 100 μm

**Fig. 8:**
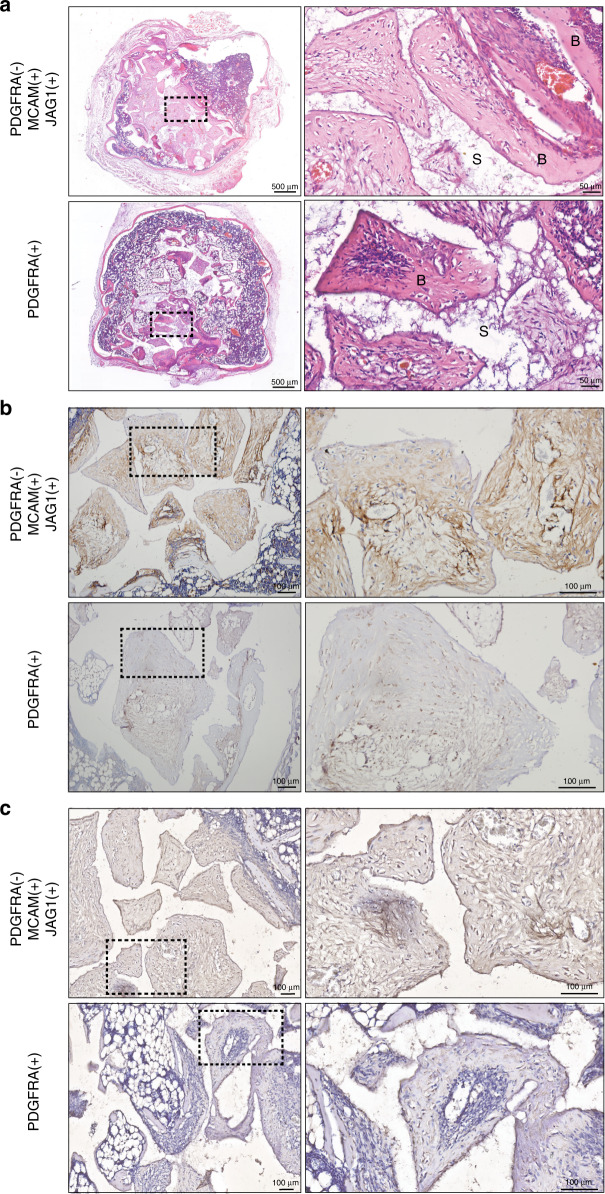
Osteogenic differentiation of MCAM( + )JAG1( + )PDGFRA(−) and PDGFRA( + ) hDPSCs in vivo. **a** Representative images of HE staining from cell-laden constructs after 4 weeks subcutaneous implantation. S scaffolds, B newly-formed bone tissues. Scale bars = 50 μm. **b** Representative images of immunohistochemical staining of human mitochondria. Scale bars = 100 μm. **c** Representative images of immunohistochemical staining of human specific human specific osteocalcin (OCN). Scale bars = 100 μm

To further explore the osteogenic and adipogenic potentials in vivo, cells from both groups were seeded on calcium phosphate scaffolds or collagen membrane, respectively, and subcutaneously implanted in nude mice for 4 weeks. MicroCT analysis revealed that cell from both MCAM(+)JAG1(+)PDGFRA(−) and PDGFRA(+) cells induced significant higher new bone formation compared to the bare scaffolds, although no statistical difference was detected between each other (Supplementary Fig. 9). Interestingly, histological analysis showed abundant bone marrow formation within the PDGFRA(+) explants, compared to the newly-formed osteoid-like structure observed from the MCAM(+)JAG1(+)PDGFRA(−) explants (Fig. 8a). Furthermore, the immunohistochemical staining human specific mitochondria (hMitochondria) and osteocalcin (hOCN) staining revealed that the implanted MCAM(+)JAG1(+)PDGFRA(−) cells actively contributed to the newly-formed bone tissue. In contrast, the majority of the newly-formed bone from the PDGFRA(-) cells was negative for hMitochondria and hOCN staining, suggesting that these cells did not directly contributed to the new bone formation (Fig. 8b, c). Similar trend was observed from histological samples for in vivo adipose tissue formation. After 4 weeks, HE staining illustrated that the MCAM(+)JAG1(+)PDGFRA(−) cells induced more adipose tissue formation compared to the PDGFRA(+) group (Fig. 7a). Furthermore, the human mitochondria-positive cells were both observed in the newly-formed adipose tissues (Fig. 7b), indicating that the transplanted MCAM(+)JAG1(+)PDGFRA(−) cells participated in the process of ectopic adipogenesis.

## Discussion

Retaining cellular identity is of great importance for maintaining functional properties of stem cells. The unstable in vivo biological efficiency of cell-based implants, owing to a lack of deep characterization of the cultured cells, has hampered their applications in tissue repair and regeneration. Recent studies have investigated the cellular composition of growing and nongrowing human teeth,^21^ but a integration linking in vivo and in vitro hDPSCs has not been elucidated. Herein, we characterized the heterogeneity of cultured and freshly isolated hDPSCs and identified the cell type shift on the single-cell level. Interestingly, although in vitro monolayer culture induces a dramatic alteration of transcriptional profiles and a clear phenotype switch, the MCAM(+)JAG1(+)PDGFRA(−) subpopulation still maintained their in vivo transcriptional signatures and revealed higher multipotent differentiation capabilities.

DPSCs are traditionally expanded in vitro prior to their basic and clinical application. However, long-term culture results in continuous changes to these cells and it is difficult to predict the dynamic changes and functional variations of subpopulations after prolonged culture duration or exposure to external factors.^19,20^ After extended in vitro culture, cells in cluster 0, 1, and 2 all displayed elevated expression of genes associated with ossification. In addition, aging was one of the enriched biological processes in cluster 0. These results are in line with the previous study, that is, MSCs undergo replicative senescence, tend to lose multipotential properties and differentiate to osteogenic cell lineage.^17^ Studies have also reported that the differentiation capability of hDPSCs was gradually reduced following cell cultivation.^18^ According to our data, though monolayer culture decreased most cell types of hDPSCs and induced an apparent phenotype switch, MCAM(+)JAG1(+)PDGFRA(−) subpopulation displayed minimal transcriptional changes and functional alterations. The attenuated potency of monolayer cultured hDPSCs may partly due to the limited fraction of cells that maintain their cellular identity and multilineage potentials. However, although the MCAM(+)JAG1(+)PDGFRA(−) hDPSCs exhibited higher multilineage differentiation abilities, the proportion of this subpopulation decreased in the long-term expansion in vitro. The gradual decline of this subpopulation may be responsible for the reduced biological function of in vitro cultured hDPSCs.

The maintenance of the cellular identity and its specific stem cell pool requires collaboration and molecular crosstalk among the heterogeneous subpopulations. Stem cells typically reside in their respective microenvironments or niches that provide signaling cues to regulate their fates and maintain tissue homeostasis.^27^ Cell interactions form networks of cytokines and growth factors. Various cell types and their secreted key factors, especially endothelial cell-secreted factors in the vascular niche, are necessary to promote self-renewal of stem cells.^28^ Our cultured data revealed that cycling cells in cluster 4, 6, and 7 support the stem cell reservoir of cluster 0–2 and 5, which might play a role in maintain the self-renewal and heterogenous of hDPSCs. In addition, cells in cluster 0 express several cytokines,^29^ like *CXCL12, KITLG*, and *ANGPT1*. These secreted factors may also contribute to the functional maintenance of the cultured hDPSC pool.

Compared to the recently published studies about scRNA-seq of human dental pulps, we found several markers they identified were also expressed in the MCAM(+)JAG1(+)PDGFRA(−) subpopulation. Pagella et al.^23^ demonstrated that MSCs in dental pulps were characterized by the higher expression of *NOTCH3*, *MYH11*, *THY1*, and *ID4*. Consistent with this study, our data also showed that the PDGFRA(−)MCAM(+)JAG1(+) subpopulation of hDPSC (cluster 4) expressed these markers (Supplementary Fig. 11a). Furthermore, PDGFRA(−)MCAM(+)JAG1(+) subpopulation of DPSC (cluster 4) also expressed the other characteristic markers of DPSCs such as *BGN*, *SOX4* and *JAG1*, which was consistent with the study of Yin et al.^22^ (Supplementary Fig. 11b). Taken together, our data was consistent with the previous studies using DPSCs obtained from different patients, which illustrated the reproducibility of our sequencing data.

In our study, the identified markers positively labeled the perivascular area, which further confirmed that the perivascular area was the niche for most types of MSCs.^26^ PDGFRA is highly expressed in many adult fibroblasts and has been assigned a mesenchymal progenitor identity, such as bone marrow resident skeletal progenitor cell population,^30^ myofibroblast progenitors,^31,32^ and fibro/adipogenic progenitors.^33,34^ PDGFRA functions as a gatekeeper of adipogenesis and distinct PDGFRA-marked perivascular cell subpopulations possess different osteogenic potentials within adipose tissue.^35^ We observed that PDGFRA(+) expressing cells were also present in perivascular locations and displayed weaker multiple differentiation potentials. In addition, the number of PDGFRA(+) cells increased dramatically after in vitro expansion but PDGFRA was rarely detected in freshly isolated dental pulp cells. It indicated that the cell surface marker expression profiles differ from their in vivo environment and the upregulation expression of PDGFRA might infer the cellular phenotypic switch. Some cells in the cell-cycle phase also expressed PDGFRA, consistent with the previous observation that PDGFRA(+) stromal cells proliferate faster than their PDGFRA(−) counterparts.^34^ MCAM/CD146 is primarily used as a biomarker of endothelial lineages and plays important roles in cell growth, migration, and angiogenesis.^36^ MCAM is closely associated with the multipotency, proliferation, and stemness of MSCs.^37–39^ MCAM may regulate cellular senescence of human umbilical cord blood-derived MSCs.^40^ Cells with high levels of MCAM were identified as human pericytes that ensheathed the vasculature of multiple organs. Presently, MCAM and PDGFRA were expressed differently in two separate partitions. Although they are both thought to be markers for multipotent progenitor cells, the trilineage differentiation capabilities of MCAM(+) JAG1(+)PDGFRA(−) cells were stronger than that of PDGFRA(+) cells in vitro. A similar result was reported previously, in which MSCs expressing distinct levels of MCAM and PDGFRA differed in their establishment in the supporting niche and in the functional maintenance of HSCs.^41^ Other marker genes, like NOTCH3, THY1 and ITGA1 also expressed in MCAM(+) JAG1(+)PDGFRA(−) hDPSCs. Our in vivo osteogenic findings also showed similar results with Yasui et al.^42^ who found that THY1 (high+) hDPSCs promote new bone formation using lineage tracing.

Like other tissues, dental pulp undergoes age-associated changes, such as decreases in volume and vascularization, and modifications in the biological characteristics of DPSCs. Differentiation potentials and proliferative capabilities of DPSCs were reportedly decreased in aged donors.^43^ We observed a slight reduced MCAM(+)JAG(+)PDGFRA(−) cells in the aged group but this change was not obvious. This result did not contradict previous conclusion of an age-dependent decline of the stem cell niche in dental pulp.^43^ Because the total number of stem cells may decreased but the percentage of MCAM(+)JAG(+)PDGFRA(−) cells stayed stable. Inflammation is a common cause of the destruction of the dentin-dental pulp complex. Inflammation can activate and promote DPSC proliferation and differentiation to odontoblasts or fibroblasts in response to injury.^44,45^ The present observations revealed insignificantly increased numbers of MCAM(+)JAG(+)PDGFRA(−) cells, suggesting that the dental caries may evoke the proliferation and differentiation of different subpopulations of hDPSCs. Due to the heterogeneity of hDPSCs, other subpopulations may take a part in this process. Other reason for the lack of significant difference between normal and dental caries groups may result from the asymmetric division of stem cells. Inflammation stimulates stem cells to produce two daughter cells, one is similar to its mother cell and maintains the original stem cell pool, the other differentiate into downstream cell types. However, detailed mechanisms and whether aging and inflammation affect the proliferation and differentiation potentials of MCAM(+)JAG(+)PDGFRA(−) cells need further explored in the future.

In summary, our findings revealed the heterogenous cell subpopulations in fresh and cultured hDPSCs and a cellular composition switch during in vitro expansion. Interestingly, one subpopulation characterized as MCAM(+)JAG(+)PDGFRA(−) maintained the transcriptional characteristics as the fresh isolated DPSCs. Specifically, the MCAM(+)JAG(+)PDGFRA(−) subpopulation located in the perivascular niche and the expression level remained stable in dental pulps regardless of aging or inflammation conditions. Furthermore, the MCAM(+)JAG(+)PDGFRA(−) subpopulation appeared higher proliferation and trilineage differentiation capabilities. Taken together, our data revealed that in vitro monolayer culture significantly altered the hDPSCs transcriptional characteristics and biological performance, which may affect their effective use in regenerative medicine.

## Materials and methods

### Primary cell cultures

The entire protocols were conducted in accordance with the Ethics Committee of the School of Stomatology, Wuhan University. The isolation of human dental pulp cells (hDPSCs) was performed as our previous study.^46^ Briefly, immediately after patients’ healthy third molars (aged 15–26) were extracted, the samples were transported to ice-cold phosphate-buffered saline (PBS, Hyclone, Logan, UT) with 100 U·mL^–1^ penicillin/streptomycin (PS, Hyclone) and rinsed repeatedly. The dental pulp tissues from five individual patients were then softly removed, pooled together, cut into tiny pieces and digested by 3 mg·mL^–1^ type I collagenase and 4 mg·mL^–1^ Dispase II (Roche) for 1 h at 37 °C in water batch, aided by shaking every 10 min. After enzymatic dissociation, cell suspension was passed through a 40 μm Falcon Cell Strainers. The freshly obtained cell suspension was then centrifuged for 5 min at 1000 r·min^–1^. The cell pellet was then resuspended into alpha Dulbecco’s modified Eagle’s medium (α-MEM, Hyclone, Logan, UT) with 20% fetal bovine serum (FBS, Gibco) and 100 U·mL^–1^ PS, at 37 °C and 5% CO_2_ in 25 cm^2^ flasks. Cells started to expanded; and after 10 days, the cultured cells were trypsinized, rinsed and prepared for the single-cell suspension used for scRNA-seq or FACS cell sorting.

### Single-cell suspension preparation for scRNA-seq

The freshly isolated or the cultured single-cell suspension was rinsed twice with Dulbecco’s phosphate-buffered saline (DPBS, Hyclone, Logan, UT) free of magnesium and calcium. The pellets were resuspended in DPBS + 0.04% bovine serum albumin (BSA, Sigma–Aldrich) and finally filtered by 40-μm cell strainers to avoid cell aggregation. The total number of cells needed to be supplied is at least 10 times than target cell numbers. Cell concentration and viability were confirmed using a LUNA automated cell counter (Logos Biosystems). The sample consisted of >90% viable cells and the cell concentration is required to be controlled in the range of 700–1200 cells per μL. The cell debris rate and large cell clumping rate were less than 5%.

### 10× Genomics scRNA-seq library generation and sequencing

Reverse transcription and library construction were carried out following the Chromium^TM^ Single-Cell 3ʹ Reagent Kits v2 protocol (10× Genomics) according to the manufacturer’s guides. Four steps are introduced to perform: Gel Bead-In-Emulsions (GEMs) generation and barcoding, cleanup and cDNA amplification, library construction by enzymatic fragmenting and size selection, sequencing prepared libraries. Qubit 3.0 was used preliminarily for quantification after the library was constructed and the library was diluted to 1 ng·μL^–1^. Agilent 2100 was then used to detect the insert size of the library. If the insert size met expectation, the StepOnePlus real-time PCR System fluorescence quantitative PCR instrument was performed to accurately quantify the effective concentration (>10 nmol·L^–1^) of the library. The qualified libraries were sequenced on Illumina platform.

The original offline sequences (raw reads) generated from Illumina platform is processed to get high-quality sequences (clean reads) through removing low-quality sequences and connector contamination. All subsequent analysis was based on the clean reads. We have uploaded our original sequencing data to GSA-human data base (no. *HRA001320*)

### Single-cell RNA-sequencing analysis

#### Initial read alignment and quality control

scRNA-seq samples were processed and aligned to Ensembl genome GRCh38 using the Cell-Ranger 3.0.0 pipeline. The Cell-Ranger output a gene-barcode matrix which was loaded into Seurat v3.0 for scRNA-seq data analysis. Cells with high mitochondrial features (>10% of total mapped reads) were excluded from the analysis to remove the influence of dead cells. A small number of cells with unusually high or low numbers of mapped reads were also removed from the dataset, as these outliers could be duplets (expression of at most 7500 genes for in vitro *cultured* data and 5000 genes for uncultured data) or poorly-captured cells (expression of at least 1000 genes for in vitro cultured data and 500 genes for uncultured data). After these filters 8963 in vitro cultured cells/6970 uncultured cells remained (out of 10 116/7000 initially provided by the Cell-Ranger pipeline).

#### Dimensional reduction

Highly variable genes were identified and subjected to dimensional reduction by principal component analysis. The Elbow method implemented in Seurat was used to select a ranking of principal components based on the percentage of variance explained by each one. We can observe an ‘elbow’ around PC 10–12, suggesting that majority of the true signal is captured in the first 12 PCs. The Elbow plot of in vivo data indicated that PC 8 would be fine.

#### Identification of clusters

The identification of biologically relevant communities was performed by means of a graph-based clustering in Seurat through the construction of a KNN graph and posterior cell clusterization using the Louvain algorithm (resolution at 0.5). Cell clusters were visualized using uniform manifold approximation and projection plots with previously selected significant components as an input.

### Immunofluorescence staining

Human third molar samples were fixed in 4% paraformaldehyde (PFA) at 4 °C overnight and then washed twice with distilled water. After decalcified in 10% ethylenediaminetetraacetic acid (EDTA) for more than 3 month, the demineralized tooth samples were dehydrated in an alcohol gradient and embedded in paraffin. Sections of 5 μm thickness were de-waxed and rehydrated followed by antigens retrieval with gastric enzyme. The slides were blocked by 2% BSA and incubated with primary antibodies at 4 °C overnight. The following primary antibodies were diluted in PBS and used: mouse anti-MCAM (1:100) (Abcam ab85763), rabbit anti-JAG1 (1:100) (Abcam ab78479), and rabbit anti-PDGFRa (1:500) (CST AF1062). After washing in PBS, sections were incubated with secondary antibodies for 1 h at room temperature. The following secondary antibodies were diluted in PBS and used: anti-rabbit Alexa Fluor 488 (antgene ANT024), anti-mouse Alexa Fluor 594 (antgene ANT029) and anti-rabbit Alexa Fluor 647 (antgene ANT032). Afterward, sections were counterstained with 4′,6-diamidino-2-phenylindole (DAPI) to label nuclei. Stained samples were imaged with a ZEISS LSM 710 confocal laser-scanning microscope and analyzed by ZEISS ZEN microscope software.

### Flow-cytometry analysis and FACS-based cell sorting

Adequate cultured primary hDPSCs were collected, washed, filtered and finally resuspended in 2% FBS/PBS for further cell sorting.

The method of obtaining fresh human dental pulp cell suspensions were described above. After filtration and centrifugation, the cell pellets were resuspended in red blood cell lysis solution (Milteny) for 15 min at RT and then centrifugated at 300 × *g* for 5 min. Cells were washed twice with 2% FBS/PBS and filtered to obtain fresh cell suspensions for further flow-cytometry analysis.

Cell suspensions (10^5^ for analysis and 10^7^ for sorting) were then performed to multi-color immunofluorescence staining. Each of the following conjugated anti-human monoclonal antibodies were incubated with the cell suspensions at 37 °C for 30 min in the dark: MCAM/PE (BD 550315), JAG1/APC (R&D FAB1277A) and PDGFRa/BB700 (BD 746023). The data were analyzed and sorted by the FACS Calibur flow cytometer (Becton Dickinson, Franklin Lakes, NJ). PBS tube was used as blank control and single stained tubes as compensation controls. Data were analyzed using FlowJo^TM^ software (Ashland, OR).

### In vivo subcutaneous implantation

Cells were trypsinized, counted and resuspended in PBS. Afterwards, 20 μL of cell suspension were drop seeded onto the tricalcium phosphate scaffolds (RB-a-SK-005G, Shanghai, China). The loading dosage of cells (50,000 cells/mm^3^) were normalized to the open volume (28 mm^3^) and the total volume space (42 mm^3^) of the scaffolds, which were determined from the microCT-based image analysis in our preliminary experiment. After 30 min of incubation at 37 °C, 5% CO_2_, and 95% relative humidity, 2 mL of α-MEM was added. The seeded scaffolds were incubated overnight to allow cell attachment before implanted in BALB/c nude mice. Cell-laden scaffolds were randomly implanted subcutaneously in the shoulder and the back area of the cervical region of animals. After 4 weeks of implantation, the mice were sacrificed and the implants were collected for further analyses.

For ectopic adipose tissue formation, cells were cultured in the adipogenic media (Cyagen Biosciences Inc, Sunnyvale, CA) for 1 week. 2 × 10^6^ cells from each groups were typsinized, resuspended and mixed with the collagen membrane scaffold material. The seeded scaffolds were placed in a shaker at 37 °C for 2 h and then centrifuged at 150 × *g* for 5 min to allow adherence. After implanted into the subcutaneous pockets of nude mice for 4 weeks, the tissues were harvested and analyzed by staining with H&E.

### Microfocus computed tomography (microCT) and 3D image anasis

Samples are scanned using the SkyScan 1276 system (Bruker) microCT. The operation condition was 60 kV, 200 μA and a 0.25 mm filter of aluminum. 3D parametric analysis was performed within the region of interest (ROI) using CTAn software (Bruker). We calculated the bone volume in the total explant space as defined by the ROI as well as the inside scaffold volume only.

### In vitro multipotential differentiation

For osteogenic differentiation, cells were cultured in the basal 10% FBS/1%PS/α-MEM medium supplemented with 10 mmol·L^–1^ sodium β-glycerophosphate (Sigma–Aldrich), 50 mg·mL^–1^ ascorbic acid (Sigma–Aldrich), and 10 mmol·L^–1^ dexamethasone (Sigma–Aldrich). After fixation in 4% PFA for 30 min, cells were incubated with the Alizarin Red S (ARS) staining solution (Sigma) for 20 min at room temperature at day 21. For ARS measurement, the stain was extracted using 10% cetylpyridinium chloride (Sigma) overnight and then read the absorbance at 550 nm.

For adipogenic differentiation, cells were cultured in the adipogenic medium (Cyagen Biosciences Inc, Sunnyvale, CA). Lipid droplets were then stained with Oil Red O stain kit (Solarbio) according to the recommended protocol. Briefly, cells were fixed in ORO Fixative solution for 30 min and washed twice with water. Followed by immersion in 60% isopropyl alcohol, cells were covered by fresh prepared ORO Stain solution for 30 min. Washing 5 times to remove excessive staining solution and then photographed.

For chondrogenic differentiation, cell pellet culture system was used to access the chondrogenic differentiation potentials. Chondrogenic media (Cyagen Biosciences Inc, Sunnyvale, CA) consisted of 1% (v/v) ITS supplement, 0.1% (v/v) sodium pyruvate, 0.3% (v/v) absorbate, 0.1% (v/v) proline, 0.01% (v/v) dexamethasone and 1% (v/v) TGF-β3. Pellets were made by centrifugation of 5 × 10^5^ cells at 250 g for 5 min in 15 mL polypropylene tubes. Media was changed every 3 days. After 28 days, 3 pellets of each group were fixed, embedded, sectioned and then stained for 30 min with alcian blue solution 8GX. Blue staining indicated the synthesis of proteoglycans by chondrocytes. For quantification, the other three cultured pellets were fixed and stained. Then alcian blue dye was extracted with 4 mol·L^–1^ guanidine HCL (pH5.8) overnight, and its absorbance was measured at 590 nm.

### Sphere culture system

Sorted single cells (10^4^) of MCMA(+)JAG1(+)PDGFRA(−) and PDGFRA(+) hDPSCs were plated per well in a 6-well Ultra-Low attachment culture dishes (Corning) in serum-free DMEM/F12 culture medium (Hyclone) supplemented with 100 U/ml PS, 20 ng/mL epidermal growth factor (PeproTech), and 20 ng/mL basic fibroblast growth factor (PeproTech) and 2% FBS. Add some fresh medium to the spheres every 3–4 days during cultural period. The spheres formed were observed under a microscope on day 1–7. At day 7, we counted numbers of spheres that exceed 25 μm in diameter.

### Quantitative real-time PCR (qPCR) analysis

Total RNAs from primary hDPSCs were isolated using the HP Total RNA Kit (Omega bio-tech, Norcross, GA), followed by cDNA synthesis using the Revert Aid First Strand cDNA Synthesis Kit (Thermo). qPCR assay for mRNA detection was performed by the SYBR Green Kit (Roche) using the QuantStudioTM 6 Flex Real-Time PCR System (Applied Biosystems, Foster City, CA). *GAPDH* was used as internal reference. The primers used in qRT-PCR were showed in Supplementary Table.

### Western blot analysis

Cells were lysed using lysis buffer added with protease inhibitor at various time points after induction. The total protein concentrations were measured using a BCA protein assay kit (Thermo). Equal amounts f cell lysates were loaded onto a 10% SDS- polyacrylamide gel and transferred to 0.22 μm polyvinylidene fluoride membranes (Roche). After blocking with fat-free milk, the following antibodies were used: anti-RUNX2 (1:1000, Cell Signaling Technology), anti-SOX9 (1:1000, Abcam), and anti-PPARG (1:1000, Abcam). β-ACTIN was used as internal reference.

### Statistical analysis

qPCR and flow-cytometry data are presented as means ± SD. Differences were analyzed by unpaired two-tailed Student’s *t* test between two groups using Graphpad Prism 7. All measurements were from distinct biological samples. Statistical parameters are found in the figure legends, including exact *n* and number of biological repeats. Exact *P* values are shown, *P* < 0.05 was considered as significant.

## Supplementary information

Supplemental Fig1

Supplemental Fig2

Supplemental Fig3

Supplemental Fig4

Supplemental Fig5

Supplemental Fig6

Supplemental Fig7

Supplemental Fig8

Supplemental Fig9

Supplemental Fig10

Supplemental Fig11

Supplemental Fig12

Supplementary Table

## Data Availability

The data support the findings of this study are available from the corresponding author upon reasonable request.
