# Supplementary figures and images for: Single-cell characterization of monolayer cultured human dental pulp stem cells with enhanced differentiation capacity
Source: Int J Oral Sci. 2021 Dec 15;13:44. doi: 10.1038/s41368-021-00140-6 (PMC8674359; doi:10.1038/s41368-021-00140-6)

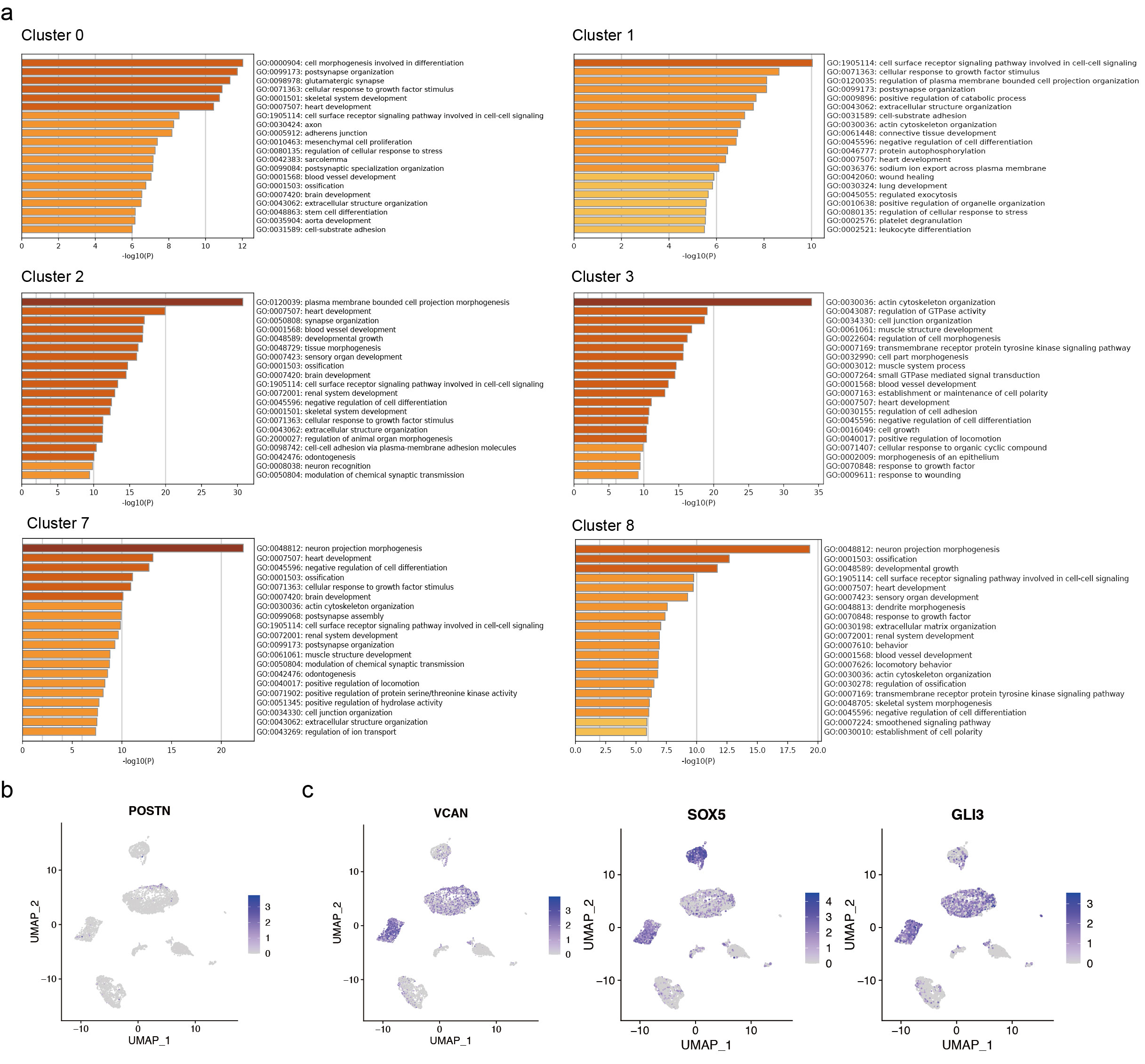

Supplement: Supplementary file 1 — Supplemental Fig1 [file 41368_2021_140_MOESM1_ESM.jpg]

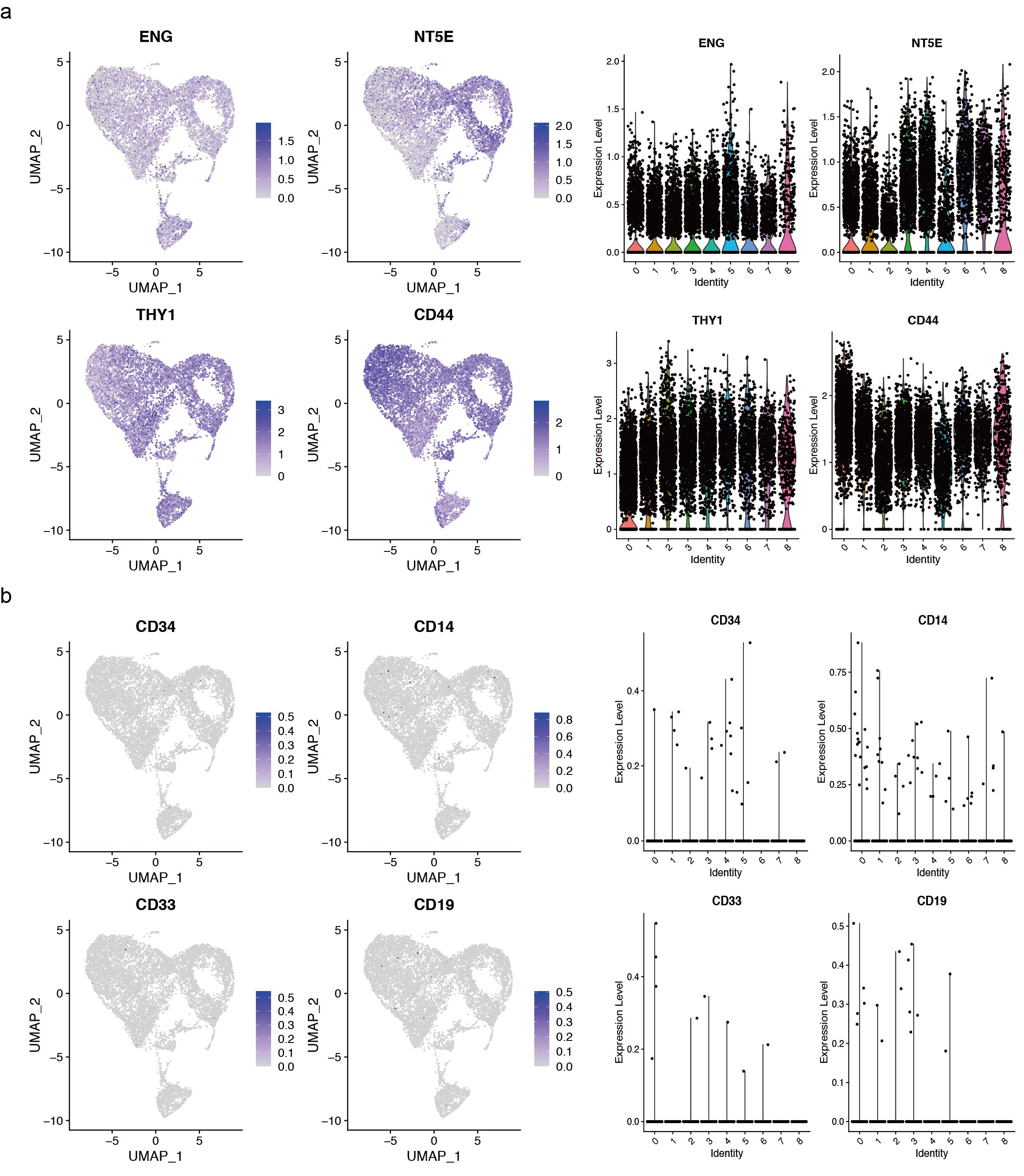

Supplement: Supplementary file 2 — Supplemental Fig2 [file 41368_2021_140_MOESM2_ESM.jpg]

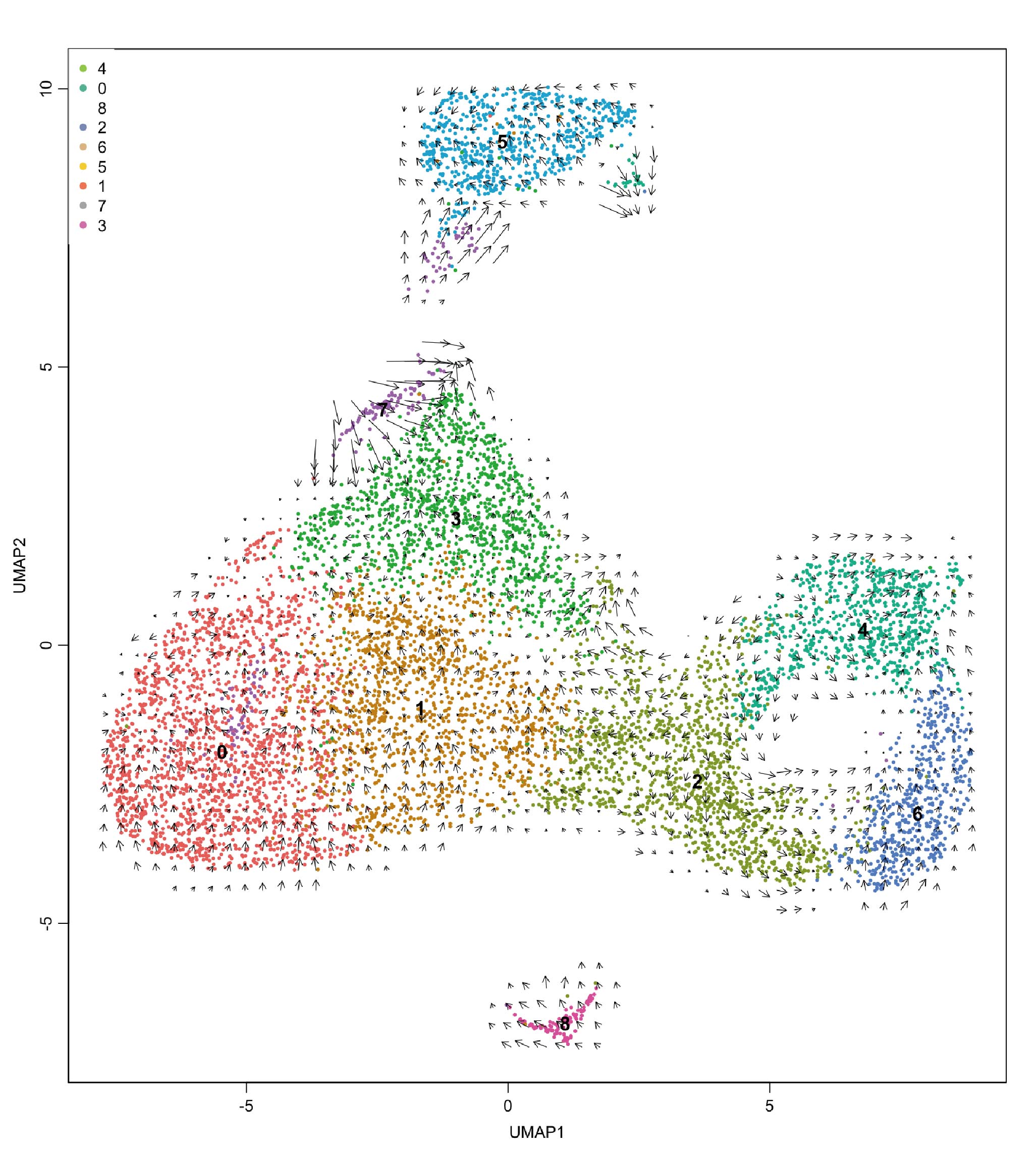

Supplement: Supplementary file 3 — Supplemental Fig3 [file 41368_2021_140_MOESM3_ESM.jpg]

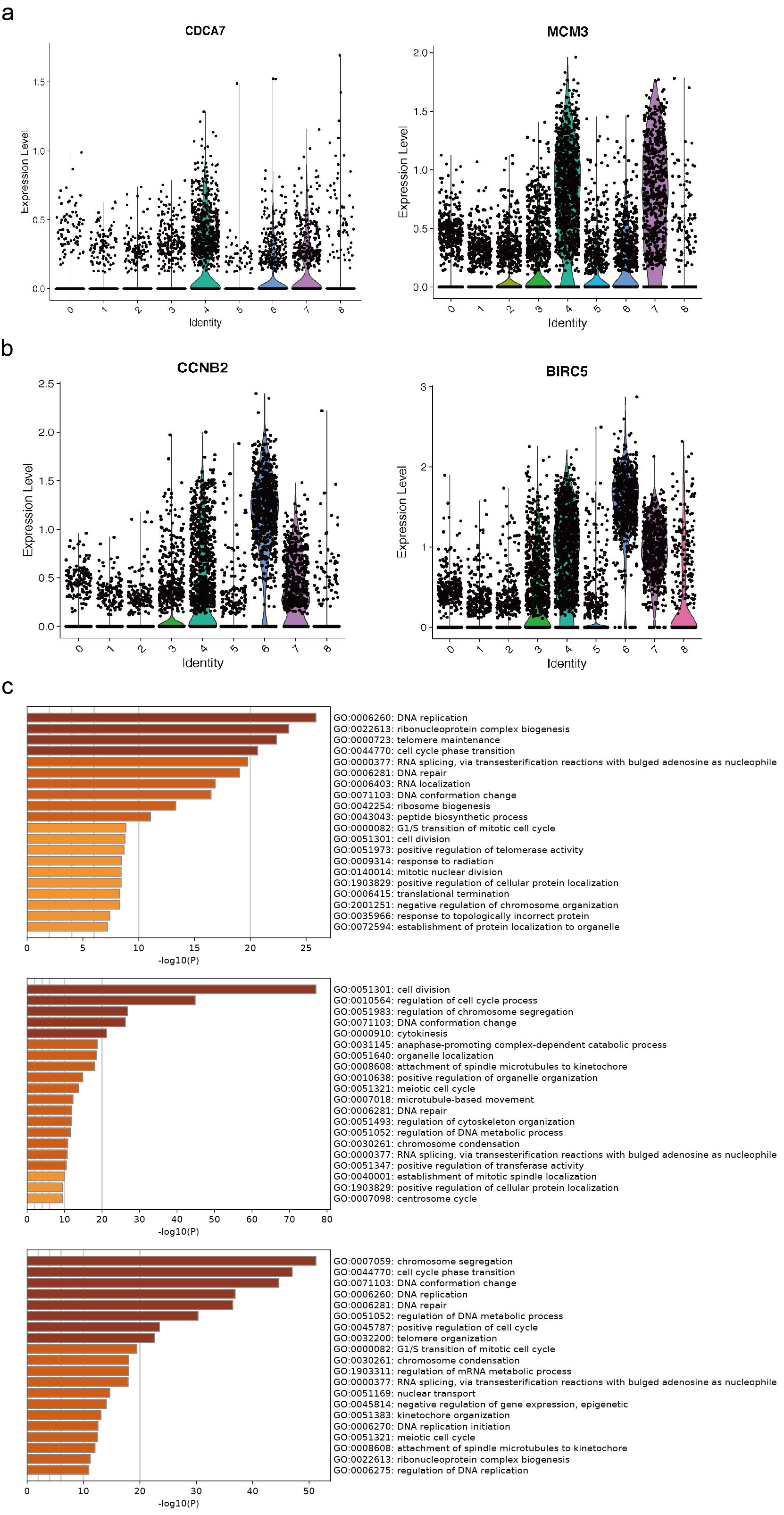

Supplement: Supplementary file 4 — Supplemental Fig4 [file 41368_2021_140_MOESM4_ESM.jpg]

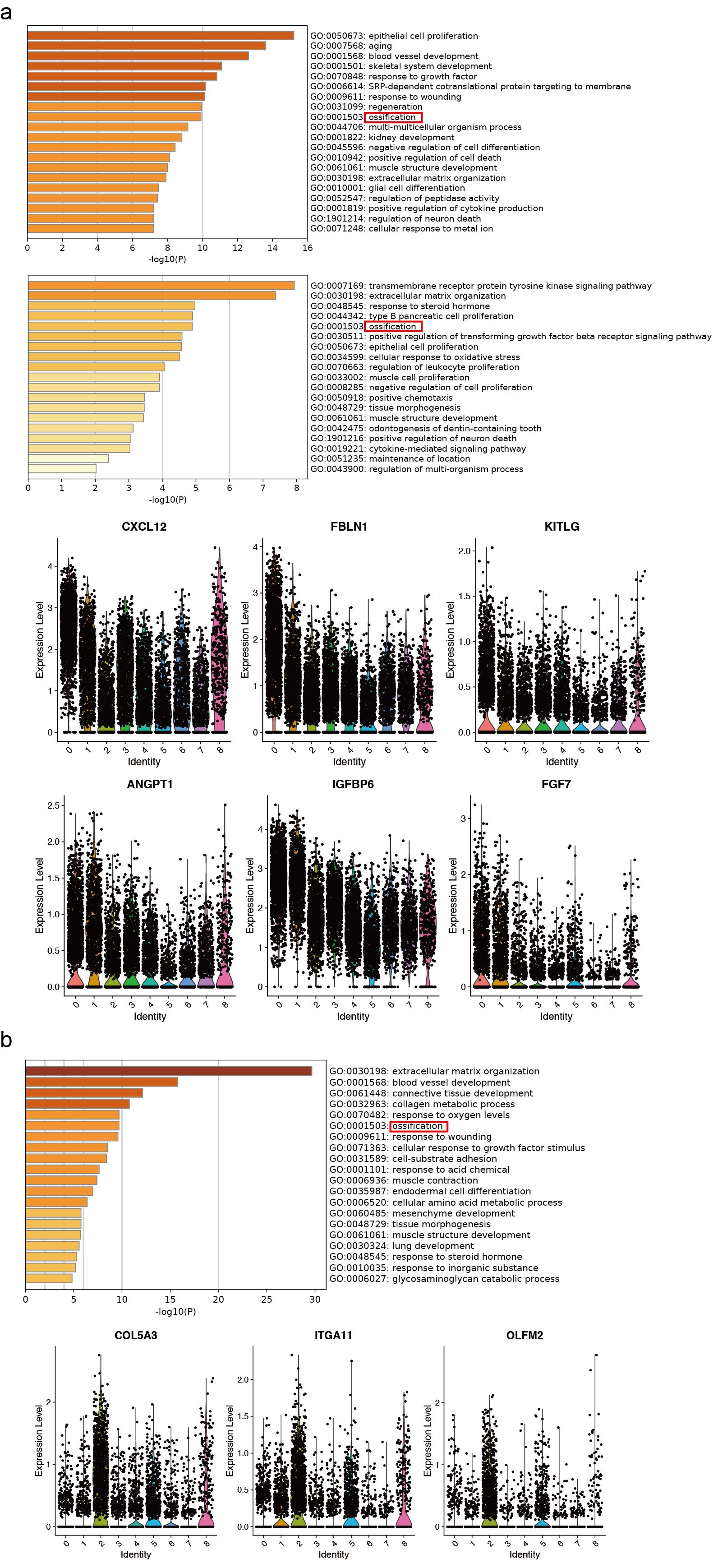

Supplement: Supplementary file 5 — Supplemental Fig5 [file 41368_2021_140_MOESM5_ESM.jpg]

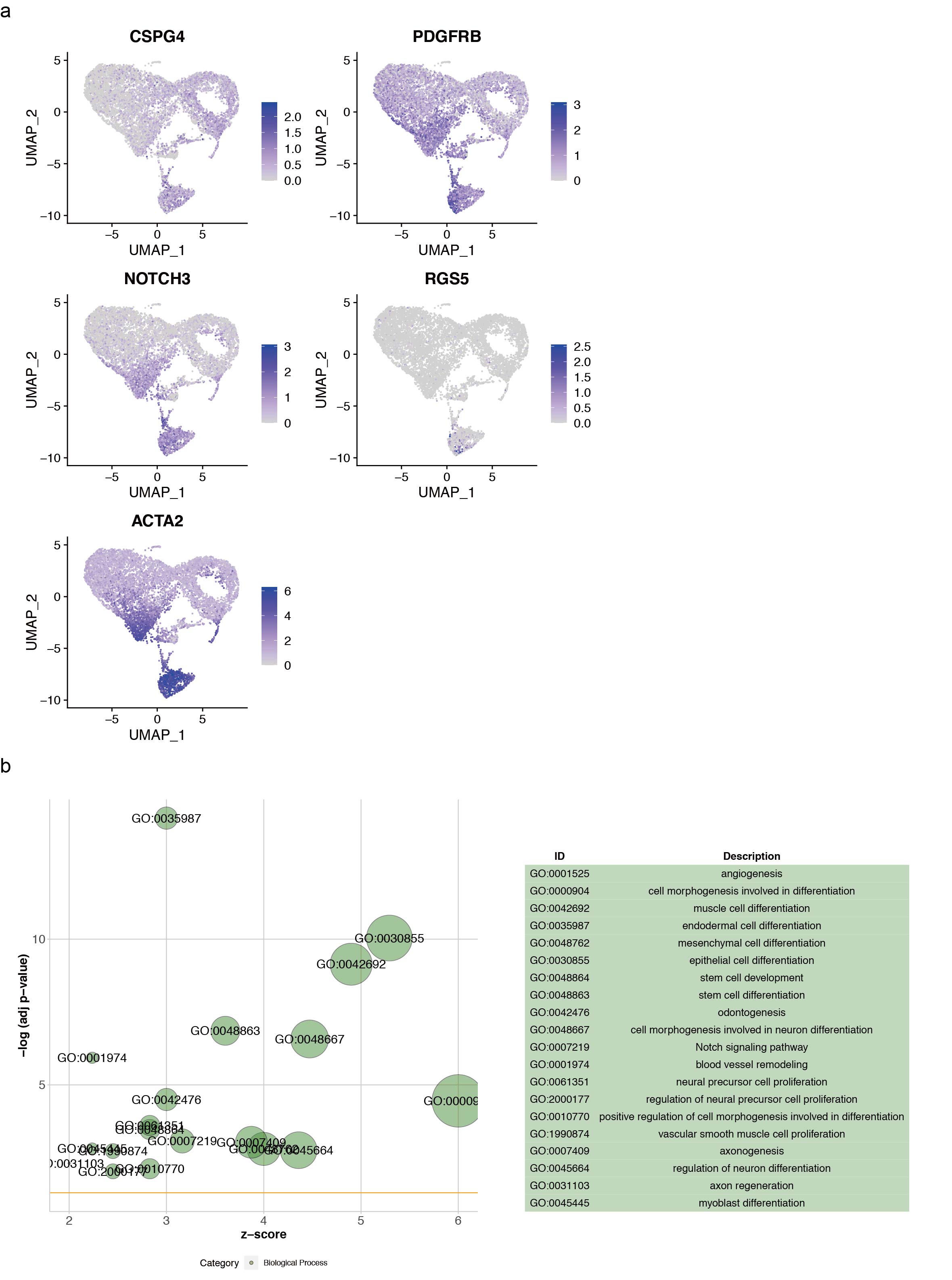

Supplement: Supplementary file 6 — Supplemental Fig6 [file 41368_2021_140_MOESM6_ESM.jpg]

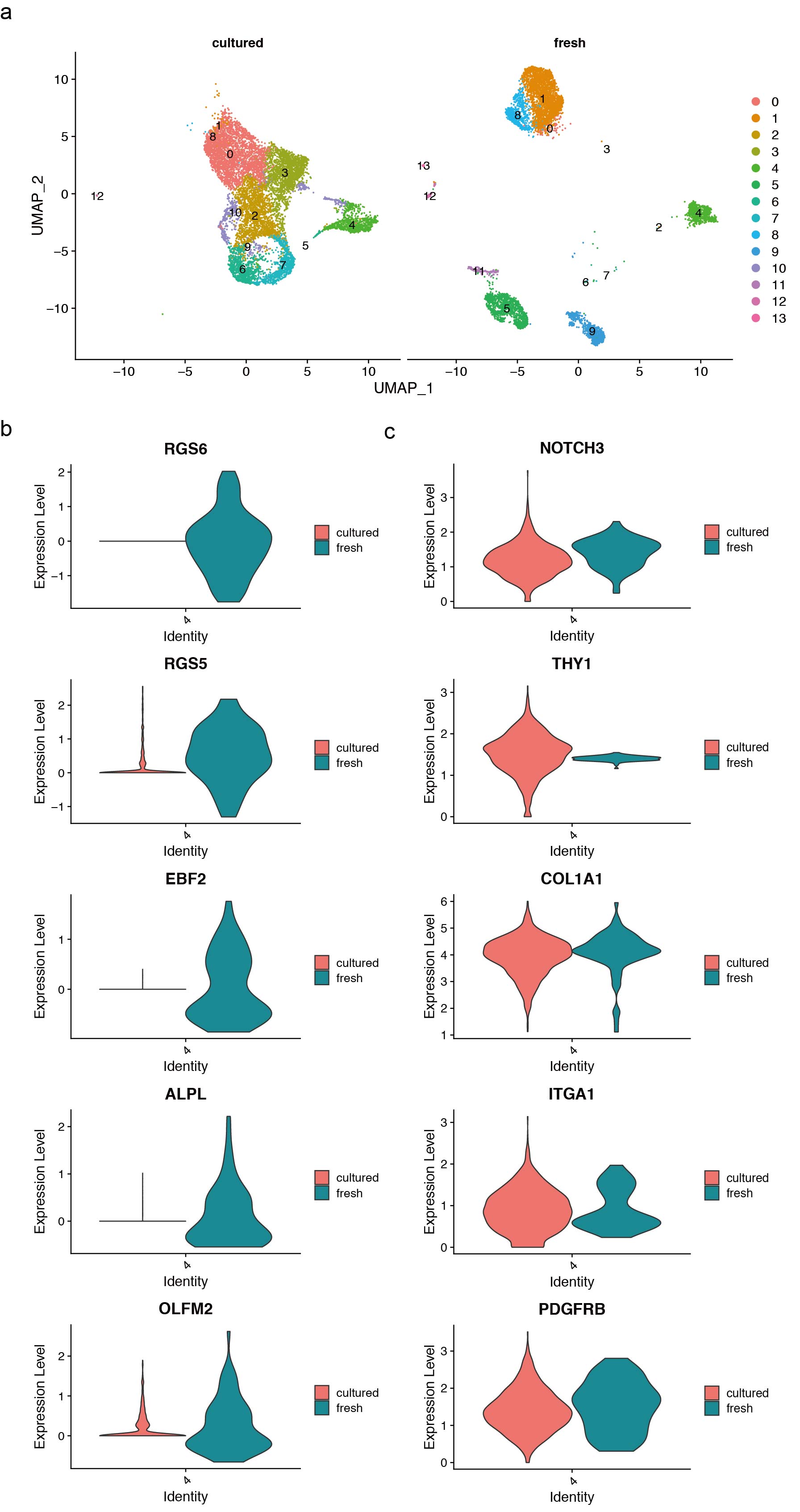

Supplement: Supplementary file 7 — Supplemental Fig7 [file 41368_2021_140_MOESM7_ESM.jpg]

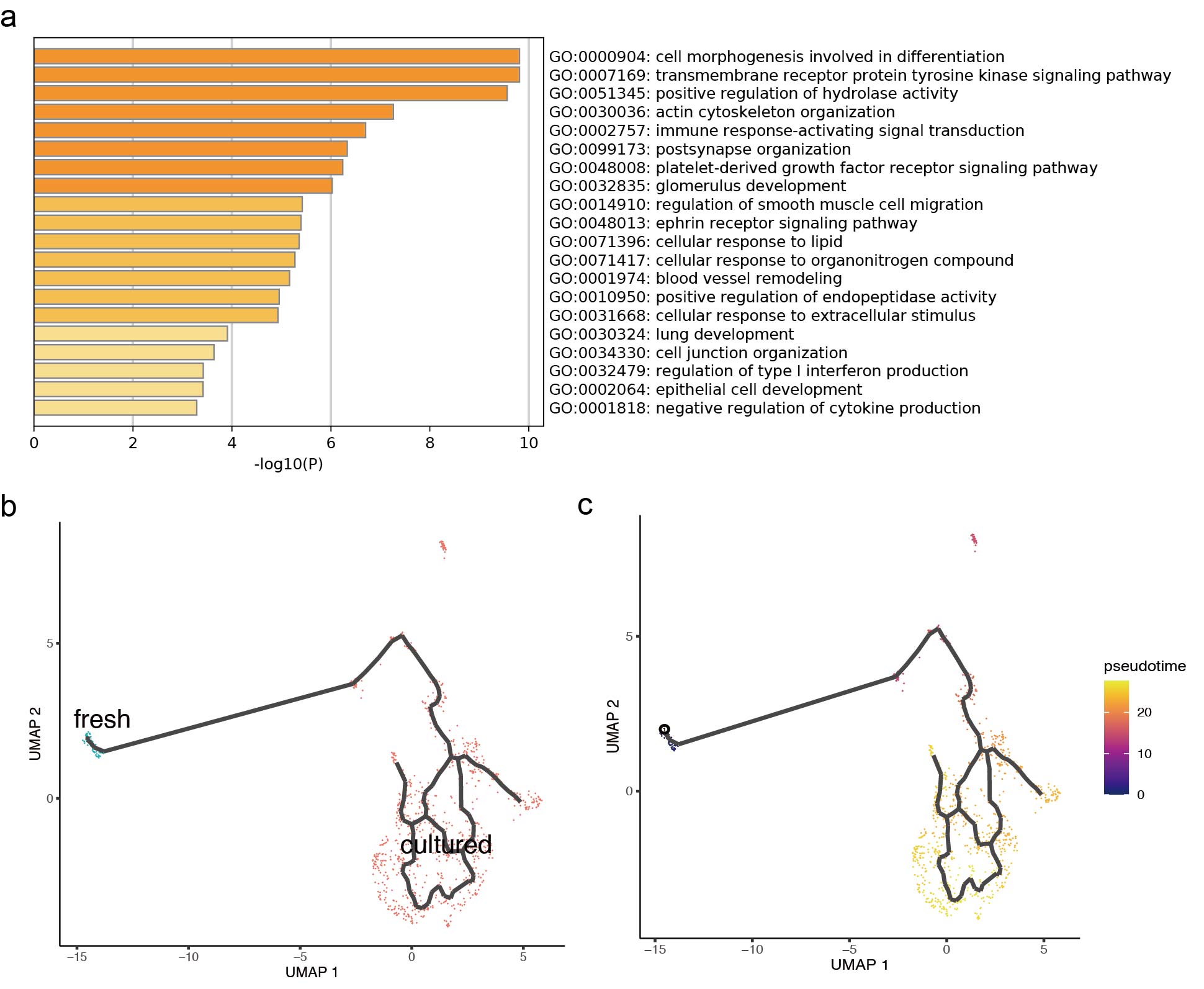

Supplement: Supplementary file 8 — Supplemental Fig8 [file 41368_2021_140_MOESM8_ESM.jpg]

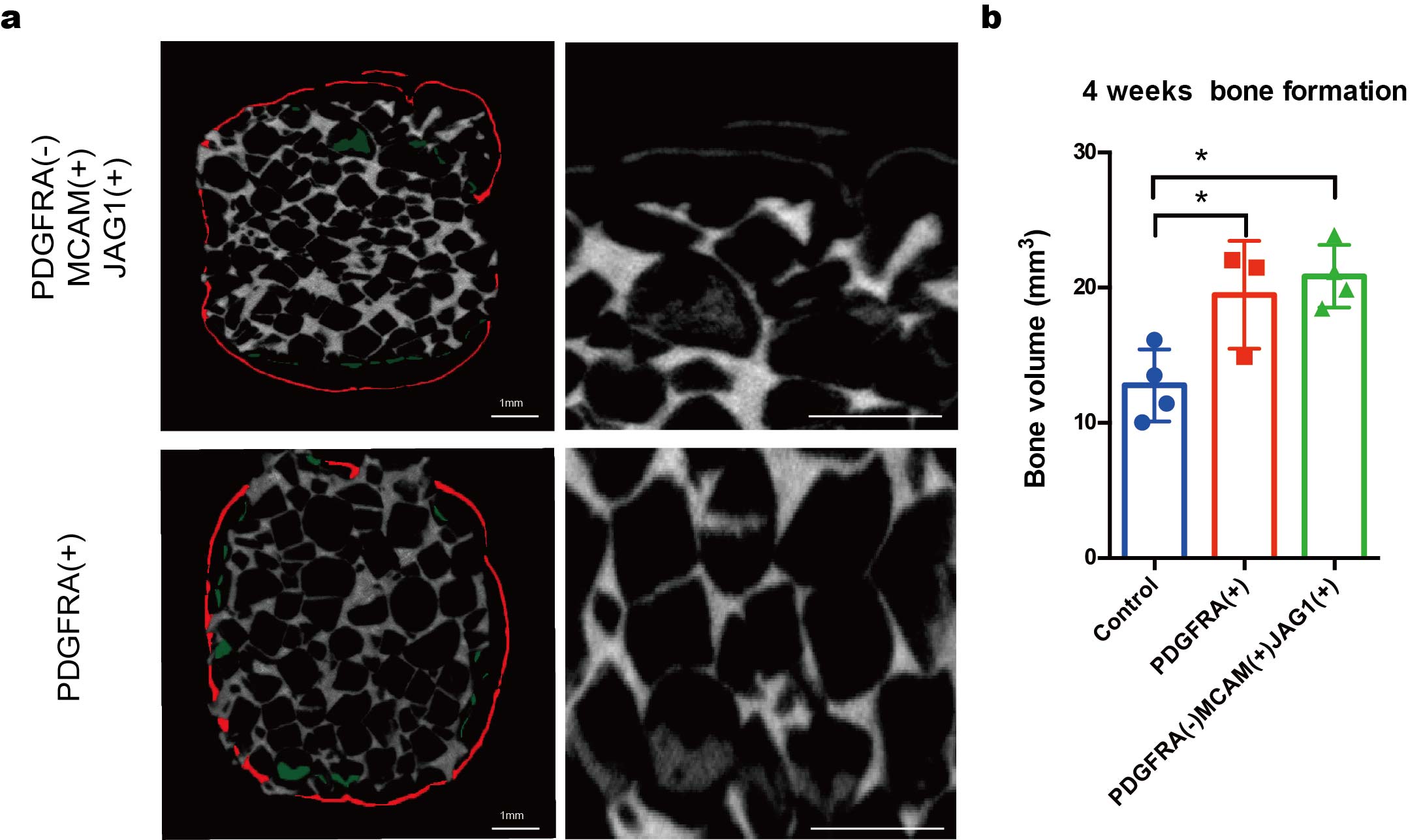

Supplement: Supplementary file 9 — Supplemental Fig9 [file 41368_2021_140_MOESM9_ESM.jpg]

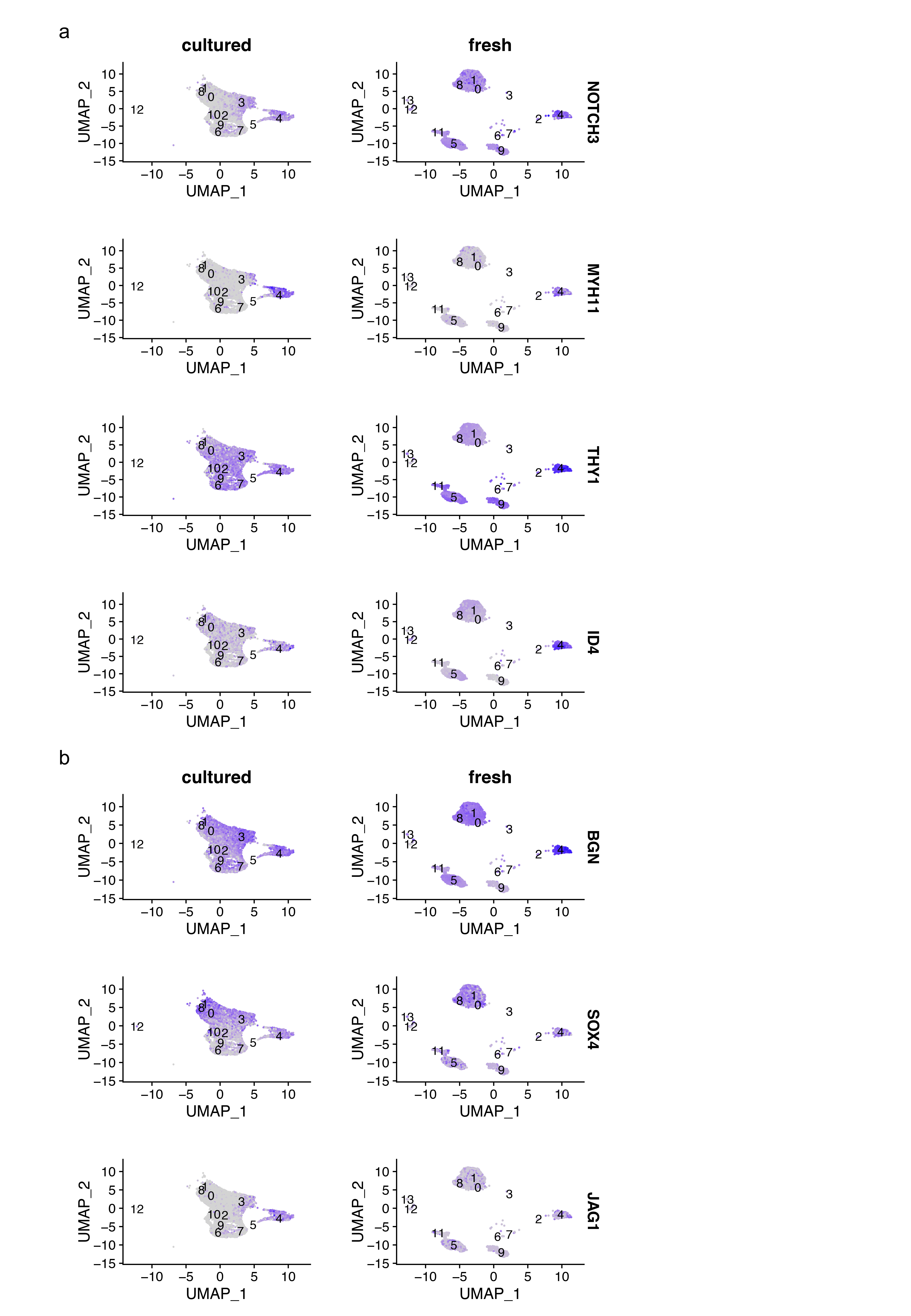

Supplement: Supplementary file 11 — Supplemental Fig11 [file 41368_2021_140_MOESM11_ESM.jpg]

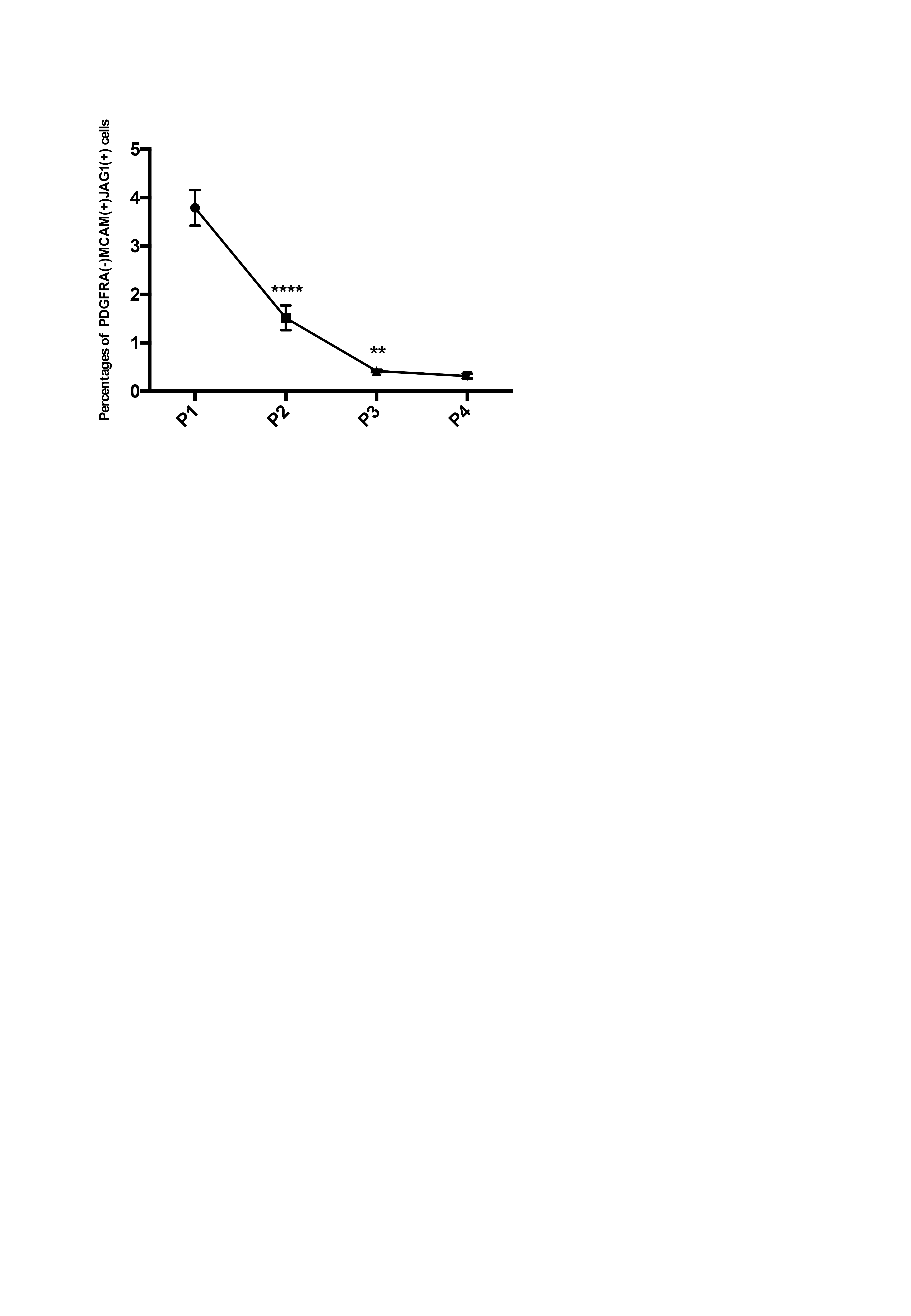

Supplement: Supplementary file 12 — Supplemental Fig12 [file 41368_2021_140_MOESM12_ESM.jpg]
