# Supplementary material for: Single-cell characterization of monolayer cultured human dental pulp stem cells with enhanced differentiation capacity
Source: Int J Oral Sci. 2021 Dec 15;13:44. doi: 10.1038/s41368-021-00140-6 (PMC8674359; doi:10.1038/s41368-021-00140-6)
Supplement: Supplementary file 13 — Supplementary Table [file 41368_2021_140_MOESM13_ESM.docx]

**Supplementary information**

**Supplementary Table. List of primers’ sequences used in this article.**

| Primer | Sequence (5’-3’) |
| --- | --- |
| *GAPDH* | Forward: TGCACCACCAACTGCTTAGC |
|  | Reverse: GGCATGGACTGTGGTCATGAG |
| *RUNX2* | Forward: CACGGAGCACAGGAAGTTGGG |
|  | Reverse: AAGATGGATTGCACGCAGGTTCTC |
| *COL1A1* | Forward: AAAGATGGACTCAACGGTCTC |
|  | Reverse: CATCGTGAGCCTTCTCTTGAG |
| *BSP* | Forward: GTCTATAGAACCACTTCCCCAC |
|  | Reverse: GCTGTACTCATCTTCATAGGCT |
| *SOX9* | Forward: GACAGCCCCCTATCGACTTC |
|  | Reverse: CAAACTCGTTGACATCGAAGG |
| *ACAN* | Forward: GATCCTTACCGTAAAGCCCATC |
|  | Reverse: CTCCAGTCTCATTCTCAACCTC |
| *COL2A1* | Forward: CAAGAACAGCATTGCCTATCTG |
|  | Reverse: GATAACAGTCTTGCCCCACTTA |
| *PPARG* | Forward: TGCTGGAGCCACAAAC |
|  | Reverse: AAACCCTATGCAACCTTC |
| *FABP4* | Forward: GGCCAAACCTAACATGATCATC |
|  | Reverse: TTATGGTGCTCTTGACTTTCCT |
| *CEBPA* | Forward: GACAAGAACAGCAACGAGTAC |
|  | Reverse: TCATTGTCACTGGTCAGCTC |
